# Denatured lysozyme-coated carbon nanotubes: a versatile biohybrid material

**DOI:** 10.1038/s41598-019-52701-9

**Published:** 2019-11-12

**Authors:** Marialuisa Siepi, Giuliana Donadio, Principia Dardano, Luca De Stefano, Daria Maria Monti, Eugenio Notomista

**Affiliations:** 1 Department of Biology, University of Naples Federico II, Via Cintia, 80126 Naples, Italy; 2 Institute for Microelectronics and Microsystems, Unit of Naples, National Research Council, Via P. Castellino 111, 80131 Naples, Italy; 3 Department of Chemical Sciences, University of Naples Federico II, Via Cintia, 80126 Naples, Italy

**Keywords:** Carbon nanotubes and fullerenes, Bioinspired materials, Biomaterials - proteins

## Abstract

Carbon nanotubes (CNTs) are among the most versatile nanomaterials, but their exploitation is hindered by limited dispersibility, especially in aqueous solvents. Here, we show that AP-LYS, a highly cationic soluble derivative of denatured hen egg lysozyme, is a very effective tool for the unbundling and solubilisation of CNTs. AP-LYS proved to mediate the complete and stable dispersion of CNTs at protein: CNT ratios ≥1: 3 (w:w) in very mild conditions (10–20 minutes sonication in ammonium acetate buffer, pH 5.0). Electrophoretic mobility and ζ-potential measurements confirmed that dispersed CNTs were coated by the protein, whereas molecular docking was used to study the interactions between AP-LYS and CNTs. AP-LYS-coated CNTs proved to be a very effective microbial cell-flocculating agent with an efficiency similar to that of chitosan, one of the best available flocculating agents, thus suggesting that this hybrid could find industrial applications in the treatment of wastewaters contaminated by microbial cells, or to remove microbial cells after fermentation processes. Moreover, we exploited the low stability of AP-LYS-coated CNT dispersions in eukaryotic cell culture media to prepare scaffolds with an extracellular matrix-like rough surface for the cultivation of eukaryotic cells.

## Introduction

Carbon nanotubes (CNTs) show outstanding mechanical, thermal and conductive properties, thanks to their high surface area, high mechanical strength, feature-rich electronic properties and excellent chemical and thermal stability^1^. These properties render CNTs appealing in different technological fields and they have been used in conductive composites, energy storage and energy conversion devices, (bio)sensors, field emission displays and radiation sources, hydrogen storage media and nanometer-sized semiconductor devices^1–5^. Moreover, biological macromolecules can be combined with CNTs to develop new hybrid molecular assemblies^6–9^. For example, in bioengineering and tissue engineering, CNTs arouse interest for the production of biomimetic scaffolds, due to their similarities with the extracellular matrix (ECM)^10,11^.

Noticeable, one of the most important prerequisites for the technological exploitation of CNTs is the formation of stable dispersions of debundled nanotubes which represents a major challenge^9,12^. Chemical functionalization of the CNT surface is a very effective technique allowing both to improve dispersibility and to functionalize CNTs. For example, Borzooeian and colleagues, through the well-known carbodiimide chemistry, bound native hen egg lysozyme (LYS) to the surface of CNTs oxidized to generate free carboxylic groups^13^. This procedure allowed to retain more than 80% of the lysozyme catalytic activity thus providing CNTs with antibacterial (lytic) activity. Unfortunately, chemical modification usually requires harsh conditions and produces defective sites on the CNT walls, with consequent impairment of their mechanical, optical, electrical, and thermal properties^14–18^. Non-covalent approaches are particularly attractive as they preserve CNT properties while improving their dispersibility^12,19^. Non-covalent strategies are generally based on the use of ultrasounds in the presence of a wide variety of water-soluble amphipathic (macro)molecules which, upon binding on the hydrophobic carbon surface, create a hydrophilic layer which will prevent their re-association. Detergents, such as SDS and Tween, are cheap and effective but show low biocompatibility. For this reason, the use of biomacromolecules such as polysaccharides, lipids, proteins and nucleic acids has attracted a special interest^9,20–22^. In addition to an improved biocompatibility, biomacromolecules often provide the opportunity to functionalize the carbon surface, e.g. through the addition of enzymes or high specific binding modules (antibodies, cell attachment domains etc.)^20,23^.

In this context, it has been shown that proteins can effectively disperse carbon nanomaterials^20,24^. Solvent-exposed hydrophobic amino acids and in particular aromatic amino acids (tryptophan, tyrosine and phenylalanine) bind to the hydrophobic carbon surfaces, thus mediating the adsorption of proteins to the carbon material^24–28^. However, some authors suggested that incubation of native proteins with CNTs could induce at least a partial denaturation, thus exposing residues normally present in the hydrophobic core^29^. For example, several studies on the interaction between native LYS and CNTs suggested that binding is reversible and does not cause significant alterations of the secondary and tertiary structure of the enzyme which, in fact, maintains most of its initial catalytic activity^30–32^. In particular, studying the X-ray structure of LYS, Nepal and coworkers hypothesized that binding would be mediated by solvent exposed clusters of hydrophobic residues^30^. The same authors also pointed out the presence of a small – presumably denatured – fraction of protein irreversibly bound to CNTs. Moreover, it is well known that some proteins, e.g. fungal hydrophobins, undergo major rearrangements, including the exposure to the solvent of large hydrophobic patches, when they bind to hydrophobic surfaces^33–35^.

On the basis of these observations we hypothesized that denatured proteins should bind more effectively to carbon surfaces than native proteins thus being better coating agents. We recently, showed that native LYS can be converted to an essentially unfolded, yet very soluble, protein by reducing the four disulphides, essential for protein stability, and blocking the resulting eight free cysteine residues with 3-bromopropylamine. This procedure not only exposes the hydrophobic residues but also adds a charged aminopropyl group to each cysteine, thus further increasing the high positive charge of native LYS. The resulting denatured modified LYS, named AP-LYS (aminopropyl-LYS), is unstructured in water but prone to recover a significant amount of secondary structure in low polarity environments^36^. AP-LYS proved to be a very effective dispersing agent for C60 fullerene, an allotropic form of carbon with very low solubility, and to allow the efficient exfoliation of the popular 2D-materials molybdenum disulfide and graphene^37^. Here, we show that AP-LYS is able to promote the debundling of multiwalled CNTs (MWNTs) as well, providing very stable dispersions of positively charged CNTs. This cationic coating provides unusual properties to nanotubes. For example, we demonstrated that AP-LYS coated MWNTs, even at very low concentrations, are able to mediate the flocculation of large amount of microbial cells (bacteria and fungi), thus rendering this bioconjugate a very promising tool for water treatment (drinking and waste), but also for the removal of cells after industrial fermentation processes. On the other hand, AP-LYS coated MWNTs are not toxic for human cells and precipitate in eukaryotic cell culture media, thus suggesting a possible use for the preparation of scaffolds for tissue engineering.

## Results and Discussion

### Preparation and characterization of MWNT dispersions

**Figure 1:**
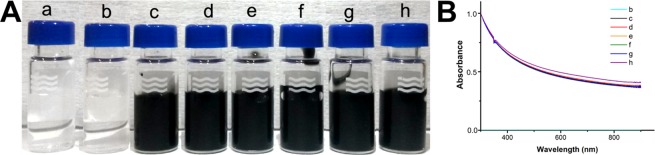
Effect of AP-LYS concentration on the debundling of MWNTs. (**A**) pictures of samples “a” to “h” (AP-LYS:MWNTs ratios and absolute concentrations of AP-LYS are reported in Table 1). (**B**) UV-Vis spectra of MWNTs samples shown in panel A.

The ability of AP-LYS to disperse MWNTs was inspected by sonicating a given amount of MWNTs (typically 3 mg of powder in a final volume of 3 mL) in the presence of increasing concentrations of AP-LYS (from 0.25 to 4 mg/mL) in 10 mM ammonium acetate (AMAC) pH 5.0 for 60 min in an ice-bath, as reported in Table 1. At the end of the sonication, all dispersions were centrifuged at 1000 g for 10 min at 4 °C to remove the undissolved material. As expected, in the absence of AP-LYS, sonication failed to provide stably dispersed MWNTs (sample “a”). As shown in Fig. 1A, in the presence of the lowest concentration of AP-LYS (0.25 mg/mL, sample “b”), the supernatant appeared clear and colorless, identical to the buffer, thus indicating that at this ratio AP-LYS is not able to disperse MWNTs, whereas, at all the ratios AP-LYS:MWNTs above 0.25:1 (samples from “c” to “h”), no sediment was found after centrifugation, and the supernatants appeared black, homogeneous, without any visible aggregate in suspension.

**Table 1:** MWNTs dispersions analyzed in this study.

sample name	[AP-LYS] (mg/mL)	MWNTs (mg of powder/mL)	AP-LYS: MWNTs (w:w)	[MWNTs]_final_^a^ (mg/mL)
a	0	1	—	0
b	0.25	1	0.25:1	0
c	0.33	1	0.33:1	1
d	0.5	1	0.5:1	1
e	1	1	1:1	1
f	2	1	2:1	1
g	3	1	3:1	1
h	4	1	4:1	1

^a^Final concentration of MWNTs in solution after removal of undissolved material.

The MWNTs/AP-LYS dispersions (samples “b”–“h”) were characterized by UV-Vis absorption spectroscopy, as unbundled CNTs strongly absorb in UV-Vis region. The spectra of the dispersions were similar to those reported in literature for MWNTs dispersions (Fig. 1B).

Very interestingly, with the exception of the sample containing 0.25 mg/mL of AP-LYS (sample “b”), all the other samples showed the same absorbance at 600 nm, thus indicating that all the dispersions contained the same amount of carbon material in solution. This result was in agreement with the absence of pellets after the centrifugation step and suggested that MWNT dispersion was an all-or-nothing process, with a critical AP-LYS:MWNTs ratio between 0.25:1 and 0.33:1. On the basis of these findings we assumed that the concentration of MWNTs in all the samples from “c” to “h” was 1 mg/mL.

All the MWNTs dispersions proved to be very stable over time being essentially unchanged after storage at 4 °C for two years (Fig. S1).

**Figure 2:**
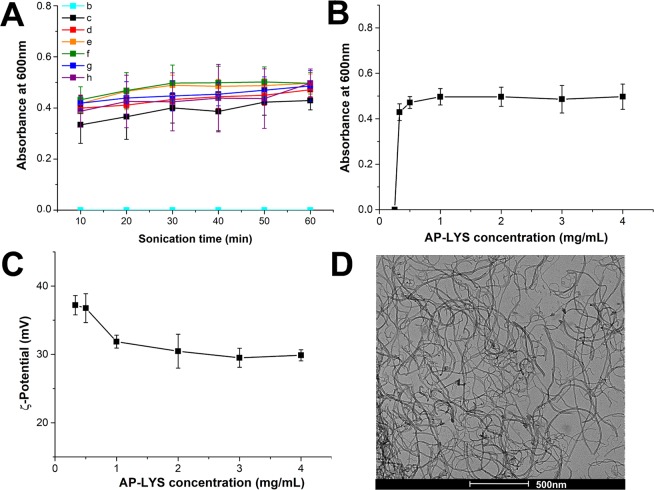
Characterization of MWNT dispersions. (**A**) Absorption at 600 nm of the MWNT dispersions reported as a function of the sonication time. (**B**) Absorbance at 600 nm of MWNT dispersions reported after 60 min sonication as a function of AP-LYS concentration. (**C**) ζ-potential of MWNT dispersions reported as a function of AP-LYS concentration. (**D**) Representative TEM image of MWNTs dispersion “c” (AP-LYS:MWNTs ratio = 0.33:1).

To study the kinetic of the dispersion process, MWNTs powder was sonicated in presence of different AP-LYS concentrations in 10 mM AMAC pH 5.0 and the absorbance at 600 nm was monitored every 10 min. Very interestingly, we found that the dispersion process is essentially complete after 10–20 min of sonication (Fig. 2A). As previously observed, increasing the amount of AP-LYS:MWNTs ratio did not increase the yield of dispersed MWNTs (Fig. 2B).

Samples from “c” to “h” were characterized by electrophoretic mobility and ζ-potential. All samples migrated toward the negative electrode and the electrophoretic mobility varied from 2.92 ± 0.11 μm s^−1^ cm V^−1^ in the sample “c” to 2.34 ± 0.06 μm s^−1^ cm V^−1^, in “h” (Fig. S2). Furthermore, all dispersions showed a positive ζ-potential (Fig. 2C), in the range +30/+37 mV. As the ζ-potential of MWNTs in distilled water is −13 mV^38^, this finding suggests that MWNTs were coated by AP-LYS. Furthermore, ζ-potential values higher than +30 mV indicated that the dispersions were stable and not prone to aggregate^39,40^.

Finally, Transmission Electronical Microscopy (TEM) analyses were performed to inspect the morphology of the samples. As shown in Fig. 2D, TEM images revealed that AP-LYS-coated MWNTs were well dispersed and unbundled.

We then evaluated the ability of AP-LYS to disperse MWNTs at different pH values and in the presence of salt. To this purpose, AP-LYS-coated MWNTs (samples “c”–“h”) were prepared at low pH and ionic strength (10 mM AMAC pH 5.0), and then diluted in 10 mM MOPS or 10 mM NaP pH 7.4 in the presence or absence of 150 mM NaCl. Samples were diluted in the appropriate buffer at different nanotube concentrations, from 0.016 to 0.25 mg/mL, centrifuged to remove aggregates, and absorbance at 600 nm was recorded and compared to that of samples diluted in 10 mM AMAC pH 5.0 (Fig. S3). We found that AP-LYS-coated MWNTs were stably dispersed in 10 mM AMAC pH 5.0 in the presence of salt (Fig. S3A), and in 10 mM MOPS pH 7.4 in the absence of salt (Fig. S3B). The presence of NaCl in MOPS buffer induced the complete precipitation of the sample “c” (AP-LYS:MWNTs = 0.33:1), and the partial precipitation of the sample “d” (AP-LYS:MWNTs = 0.5:1) (Fig. S3C). Finally, samples “c” and “d” were not stable, neither in the presence nor in the absence of 150 mM NaCl, in 10 mM NaP pH 7.4 (Fig. S3D,E). These data suggested that the stability of the AP-LYS-coated MWNTs dispersion was influenced by ionic strength, pH, buffer and AP-LYS:MWNTs ratio. Summarizing, AP-LYS-coated MWNTs dispersions were more stable at pH 5 than at pH 7.4 and, at pH 7.4, phosphate induced aggregation more efficiently that MOPS. These results were not unexpected as the net positive charge of AP-LYS, which likely contributes to the stability of the dispersions, is higher at pH 5 than at pH 7.4^36^. Moreover, the anions contained in the buffer could bind to and partially shield the positive charge of the coated MWNTs. Likely, the phosphate di-anion HPO_3_^2−^ (the main species in phosphate buffer at pH 7.4), is more effective than MOPS and chloride at performing the shielding effect.

### Molecular docking analysis

**Figure 3:**
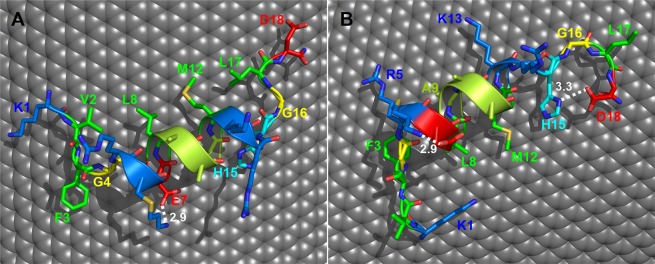
Docking of fragment 1–18 of hen egg lysozyme onto the surface of a CNT. Panels A and B show models 1 and 2, respectively. Residues are shown as sticks colored by atom type: nitrogen, blue; oxygen, red; sulfur, dark yellow; carbon atoms are colored according to amino acids properties (green, hydrophobic; light green, alanine; blue, positively charged; red, negatively charged; cyan, histidine; yellow, glycine). The carbon atoms of the surface are shown as gray spheres. Labels are shown only for the residues making significant interaction with the surface (the corresponding binding energy values are shown in Table 2). Hydrogen bonds are shown as white dotted lines and corresponding distances are in Å.

In order to try to understand the molecular basis of the interaction between AP-LYS and MWNTs we performed a docking analysis, as previously described for the interaction between AP-LYS and molybdenum disulfide (MoS_2_)^37^. We have previously shown that AP-LYS was essentially unfolded in water but it was prone to regain a significant content of helical structure in the presence of trifluoroethanol^36^, an organic solvent widely used to mimic the interaction of proteins and peptides with membranes^41–46^. By modelling the interaction of residues 1–18 of lysozyme (Fig. S4), which include the first amphipathic α-helix of native LYS (residues 4–15), we showed that this secondary structure element bound to MoS_2_ through Van der Waals and hydrophobic interactions between the hydrophobic face of the helix and the relatively hydrophobic surface of MoS_2_. In particular, we found a ΔG for the adsorption reaction of region 1–18 of about −5.3 kcal/mol (corresponding to a binding constant K = 1.7 10^3^). With a similar approach we have docked lysozyme region 1–18 to a planar graphene sheet (~74 Å × ~71 Å; 2074 carbon atoms) and to a fragment (~74 Å × ~65 Å; 2074 carbon atoms) of a carbon nanotube with an outer diameter of about 10 nm, the average diameter of the MWNTs used in this study. In the lowest energy complexes, region 1–18 formed an amphipathic helix (spanning residues 4 to 14) with the hydrophobic face in contact with the carbon surface, accordingly to the results obtained with MoS_2_. However, in the case of graphene and of the nanotube fragment, the α-helix contacted the surface with two different orientations, thus providing two alternative complexes with similar energy (Table 2, Figs 3 and S5).

**Table 2:** ΔG and binding energy values of the CNT/peptide and graphene/peptide complexes.

	CNT		Graphene	
	model 1	model 2	model 1	model 2
**ΔG**^**a**^ **(kcal/mol)**	−30.38	−29.36	−31.25	−31.42
**Total binding energy (kcal/mol)**	−45.31	−44.24	−46.57	−46.68
**Residue**	**Contributions per residue to the total binding energy**^**b**^ **(kcal/mol)**			
K1	−2.51	−1.82	−2.13	−2.78
V2	−2.97	−0.16	−3.09	−0.24
F3	−**9.58**	−**9.75**	−**10.09**	−**10.33**
G4	−0.63	<−0.1	−1.15	<−0.1
R5	<−0.1	−2.99	<−0.1	−3.06
E7	−3.36	<−0.1	−3.45	<−0.1
L8	−2.94	−2.67	−3.13	−2.59
A9	<−0.1	−1.58	<−0.1	−1.32
A11	−2.05	<−0.1	−2.06	<−0.1
M12	−4.53	−**5.63**	−4.40	−**6.18**
K13	<−0.1	−3.87	<−0.1	−4.16
H15	−**6.90**	−**5.63**	−**6.95**	−5.67
G16	−1.50	−2.74	−1.49	−2.67
L17	−4.71	−4.54	−4.53	−4.74
D18	−3.63	−2.86	−4.10	−2.94

^a^ΔG for the reaction: peptide(aq.) + carbon surface(aq.) = adsorbed peptide(aq.).

^b^The two highest contributions for each complex are in bold.

One of the two complexes was similar to that previously observed in the case of MoS_2_. In this complex, the helix was oriented so that in addition to the residues of the hydrophobic face (Leu8 and Met12) also Glu7 and Ala11 made significant contacts with the carbon surface (Table 2, Figs 3A and S5A,C). In the second complex, the helix was oriented so that in addition to residues Leu8 and Met12 also residue Arg5, Ala9 and Lys13 (on the opposite side with respect to Glu7 and Ala11) contacted the carbon surface (Table 2, Figs 3B and S5B,D). In both the orientations, residues 1–3, upstream the helical region, and residues 15–18, downstream the helical region, significantly contributed to binding (Table 2, Figs 3 and S5). Interestingly, in the case of residues 15–18, in addition to side-chains atoms, also the backbone atoms contributed to binding. This is particularly evident in the case of Gly16 (Table 2) whose peptide bonds were roughly parallel to the surface (especially in models 2), thus allowing amide-π stacking interactions as previously observed in the case of a model alanine dipeptide/graphene complex^47^. It is interesting to note that the ΔG values for the adsorption process were very similar for the complexes involving graphene and the nanotube fragment, thus indicating that region 1–18 bound with similar efficiency to both planar and curved carbon surfaces (at least with curvature similar to that examined in this study). Furthermore, all the ΔG values were considerably higher in absolute value (−29/−31 kcal/mol) with respect to that observed in the case of the MoS_2_ surface. This was an expected consequence of the higher hydrophobicity and polarizability of the aromatic carbon surfaces with respect to the MoS_2_ surface. Hypothesizing that the other amphipathic secondary structure elements present in lysozyme contribute cooperatively to the binding, this would explain the strong affinity for carbon surfaces. In general, our analysis supports the hypothesis that AP-LYS could bind to hydrophobic surfaces through amphipathic elements, at least in part corresponding to secondary structure elements of native lysozyme.

### AP-LYS-coated MWNTs as microbial cells flocculating agent

Next, as AP-LYS-coated MWNTs show a cationic nature, we inspected its flocculating ability. Indeed, flocculation is an aggregation process of suspended particles (mineral particles or microorganisms) which eventually undergo sedimentation^48^. This phenomenon can be induced by adding flocculating agents, which cause the aggregation of the particles^48^. As most inorganic particles and microbial cells are anionic, the most effective flocculating agents are synthetic or natural cationic polymers^49–52^. Thus, we incubated *Escherichia coli* cultures in the presence of AP-LYS-coated MWNTs (samples “c” and “e”) and observed an immediate formation of black flocs, visible to naked eyes, which slowly sedimented (Fig. S6). On the other hand, we did not observe significant flocculation when *Escherichia coli* cultures were incubated with AP-LYS concentrations in the range 0.1–10 µg/mL (data not shown).

**Figure 4:**
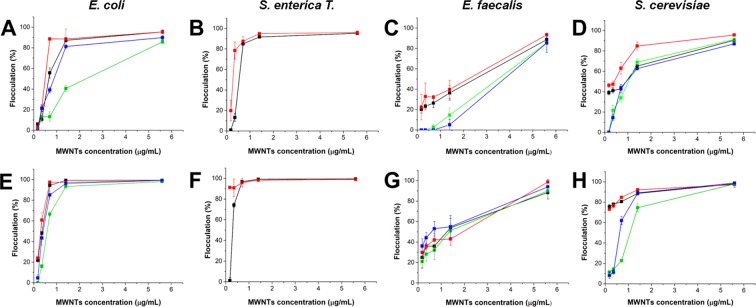
Microbial cells flocculation efficiency as function of MWNTs concentration. Flocculation of *E. coli* (**A**,**E**), *S. enterica T*. (**B**,**F**), *E. faecalis* (**C**,**G**) and *S. cerevisiae* (**D**,**H**) was measured after 2 hours (**A**–**D**) and 18 hours (**E**–**H**). In all the panels: black and green lines, flocculation induced by sample “c” in the absence and the presence of NaCl, respectively; red and blue lines, flocculation induced by sample “e” in the absence and the presence of NaCl, respectively.

In order to quantify the flocculation efficiency, increasing concentrations of AP-LYS-coated MWNTs (from 0.175 to 5.6 µg/mL) were tested on three bacterial strains which are common sweet-water contaminants, namely *Escherichia coli*, *Salmonella enterica Typhimurium* T and *Enterococcus faecalis*, and on the yeast *Saccharomyces cerevisiae*. Only dispersions “c” and “e” (AP-LYS:MWNTs ratios = 0.33:1 and 1:1, respectively) were used and incubated for 2 or 18 h with the cultures. At the end of incubation, we measured the absorbance at 600 nm of the supernatant (Fig. 4) and the flocculation percentage was calculated as described in section 4.4. The flocculation curves were obtained by plotting the flocculation % as a function of AP-LYS-coated MWNTs concentration. The flocculation assay was performed in the presence and in the absence of 150 mM NaCl to evaluate the effect of ionic strength on the flocculation efficiency.

Dispersions, “c” and “e” showed a very similar behavior. After 2 h of incubation, the efficiency of flocculation, for all the tested microorganisms, ranged between 86 and 96%, using 5.6 µg/mL of AP-LYS-coated MWNTs (Fig. 4A–D). These values increased to 89–100%, after 18 h of incubation (Fig. 4E–H). Small differences were observed after 2 h incubation in the presence of NaCl, thus suggesting that salt slows down the flocculation process. In particular, *E. coli* showed a flocculation efficiency between 87% and 95% after 2 h in the absence of NaCl using either 1.4 or 5.6 µg/mL MWNTs. On the contrary, in the presence of NaCl, 90% of flocculation was observed only using the highest amount of AP-LYS-coated MWNTs (Fig. 4A, green line). After over night sedimentation, flocculation efficiency reached 100% with both AP-LYS-coated MWNTs samples (Fig. 4E, green line). In the case of *Salmonella enterica Typhimurium* (Fig. 4B,F), a higher flocculation efficiency was found. In the absence of NaCl, 1.4 µg/mL AP-LYS-coated MWNTs were able to flocculate 95% of bacteria after 2 h (Fig. 4B), and 99% after over night incubation (Fig. 4F). The experiments with *Salmonella* were performed only in the absence of NaCl, as salt induces spontaneous flocculation of the strain (data not shown). AP-LYS-coated MWNTs (5.6 µg/mL) were able to flocculate 90% of *E. faecalis* after 2 h (Fig. 4C,G). It is intriguing to note that in the case of *E. faecalis* high flocculation efficiency was observed only after over night incubation, thus suggesting that flocculation of this strain was considerably slower than that of the others. *S. cerevisiae* had a behaviour similar to that of *E. coli*, thus demonstrating that AP-LYS-coated MWNTs could flocculate also eukaryotic cells (Fig. 4D,H). It is worth noting that AP-LYS alone, at concentrations up to 10 µg/mL, did not induce the flocculation of *E. faecalis* and *S. cerevisiae*, whereas in the case of *S. enterica* it was able to induce about 20% of bacteria flocculation after 20 h (data not shown). The last finding is not surprising as *S. enterica* cells were completely flocculated also after the simple addition of 150 mM NaCl, thus indicating that they are particularly prone to aggregate in the presence of ionic compounds.

**Figure 5:**
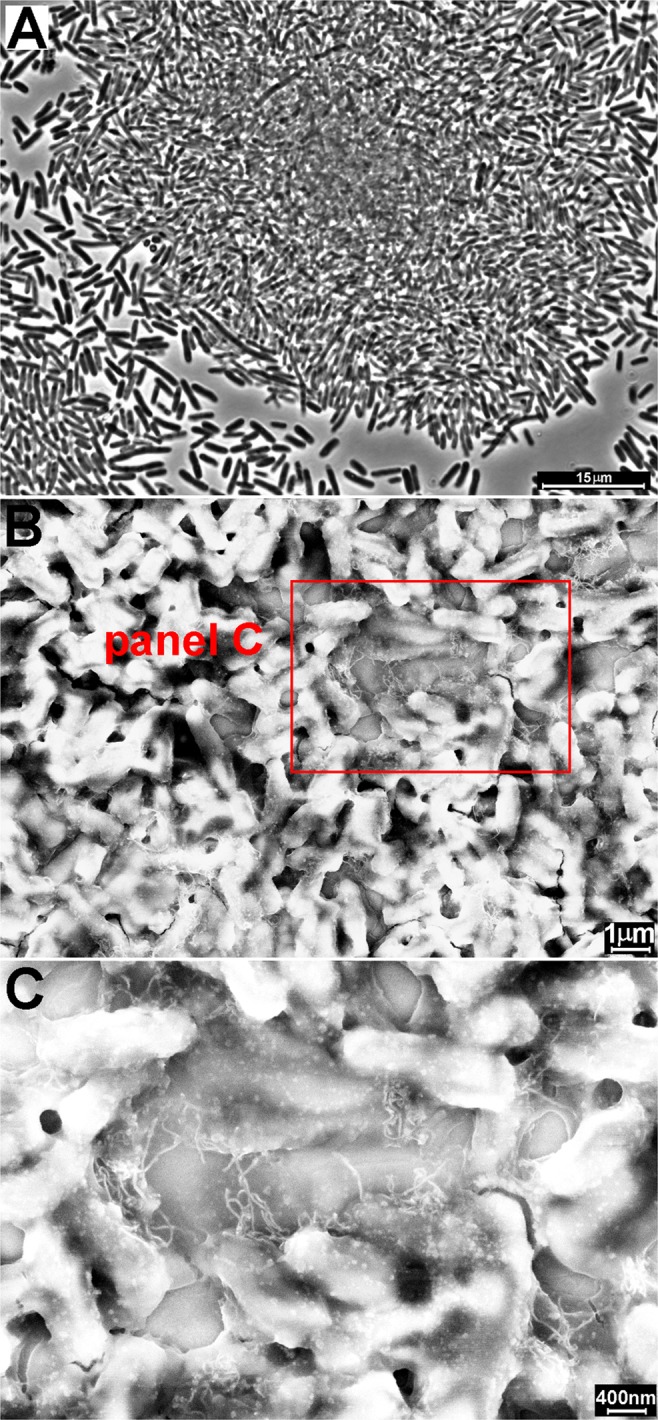
Phase contrast microscopy (**A**) and SEM (**B**,**C**) images of flocs of *E. coli* cells treated with AP-LYS-coated MWNTs dispersion “c” (AP-LYS:MWNTs ratio = 0.33:1). SEM images were obtained using an InLens detector. MWNTs are clearly visible in panel C as light strings among the cells.

The presence of flocs in the sediments was confirmed by optical microscopy analysis (Figs 5A and S7). The pellets obtained using 5.6 µg/mL of AP-LYS-coated MWNTs after 2 h of sedimentation were stained using the Live/Dead^TM^ assay and observed by a fluorescence microscope. Dead cells, due to compromised membrane, absorbed the cell impermeant propidium iodide dye and stained red, whereas cells with an intact membrane stained green absorbing the cell permeable dye SYTO 9. All the sediments contained large regular flocs (Figs 5A and S7), characterized by the presence of both live and dead cells in different ratio, depending on the analyzed strain (Fig. S7). A similar behavior was observed in the presence and in the absence of salt (not shown).

Noteworthy, a concentration as low as 0.7 µg/mL of AP-LYS-coated MWNTs was able to induce 100% flocculation of *E. coli* after over night incubation, (Fig. 4E, black and red lines), which corresponded to an effective concentration of about 0.6 mg/g of dry *E. coli* cell weight. This value is comparable or lower than those reported for partially de-acetylated-high molecular weight chitosan (MW ≥ 200 kDa), one of the most efficient cell flocculation agent described so far, which is effective at concentrations of about 0.3–5 mg/g of dry cell weight depending on the degree of chitosan de-acetylation^53–55^. The high efficiency of partially de-acetylated-high molecular weight chitosan has been explained hypothesizing that the flocculation mechanism is based not only on a nonspecific charge neutralization process caused by the adsorption of the cationic polymer at the surface of the negatively charged cells, but also on a cell “bridging” process due to specific interactions between cells and loops or tails protruding from the adsorbed polymer^54^. A similar mixed mechanism could be operating also in the case of AP-LYS-coated MWNTs. In fact, specific sequences or secondary structure elements present in the AP-LYS molecules, possibly at least in part protruding from the nanotube surface, could be able to bind specifically at the surface of bacterial cell (Fig. S8A,B). It is worth noting that human and chicken lysozymes contain antimicrobial regions similar to cationic antimicrobial peptides, a well known class of antimicrobial agents which kill bacteria by binding to and destabilizing bacterial membranes^56,57^. Taking into account the length of nanotubes – in the range 3–6 µm, i.e. comparable or even longer than that of *E. coli* cells – it can be hypothesized that AP-LYS-coated nanotubes could effectively bridge bacterial cell as schematically shown in Fig. S8B. On the other hand, free AP-LYS would not be able to induce flocculation due to its low molecular weight (about 14 kDa). In order to confirm this hypothesis, *E. coli* cells flocculated by AP-LYS-coated MWNTs were analyzed by Scanning Electron Microscopy (SEM). Samples were casted on a gold surface, dried at room temperature and imaged without metallization to exploit the high conductivity of MWNTs. Nanotubes were clearly visible as light strings among the cells in the images of both large and small cell aggregates (Figs 5B,C and S9, respectively). The presence of the nanotubes among the cells was also evidenced by AFM (Fig. S10). Therefore, both SEM and AFM confirmed that CNTs were present among bacterial cells aggregated by the addition of AP-LYS-coated MWNTs.

In addition to the flocculating ability, chitosan also possesses antimicrobial activity that depends from its positive charge. Thus, quaternization of amine groups increases the positive charge and makes it less pH sensitive, thus increasing its antimicrobial activity. It is generally assumed that chitosan kills bacterial cells by binding to their surface, damaging the integrity of bacterial membrane and causing the leakage of cells. This can be the case also for AP-LYS-coated MWNTs, in which the high charge and the presence of antimicrobial peptide-like induce surface damage and cell permeabilization. This hypothesis is sustained by propidium iodide staining after only 2 h treatment with AP-LYS-coated MWNTs.

### AP-LYS-coated MWNTs as scaffolds for eukaryotic cells

Prior to characterize the interaction of eukaryotic cells with AP-LYS-coated MWNTs, the stability of the dispersions in Dulbecco’s modified Eagle’s Medium (DMEM), one of the most widely used eukaryotic culture medium, was determined. AP-LYS-coated MWNT dispersions proved to be very unstable in DMEM, in particular in the presence of 10% fetal bovine serum (FBS). In cell culture-treated polystyrene multiwell plates filled with DMEM containing FBS, AP-LYS-coated MWNTs formed large irregular aggregates tightly bound to the bottom of the wells (images not shown). These observations prompted us to verify the possibility to use AP-LYS-coated MWNTs for the preparation of scaffolds for eukaryotic cells, as CNTs have aroused considerable interest in the field of cell culture and tissue engineering since they can form frameworks resembling extracellular matrix (ECM). For example, CNTs functionalized with ECM proteins proved to be particularly promising tools for the preparation of biocompatible scaffolds for cultivation of cell lines difficult to grow, such as stem cells, neurons, osteoblasts and myocytes^10,58^. However, handling of CNTs is made difficult by the necessity to unbundle/disperse them and, successively, to control their assembly in matrix-like insoluble structures. From this point of view the high stability at low ionic strength and moderately acidic pH values of AP-LYS-coated MWNT dispersions, along with their ability to form insoluble aggregates at physiological conditions, provide the possibility to design simple strategies to assemble scaffolds.

**Figure 6:**
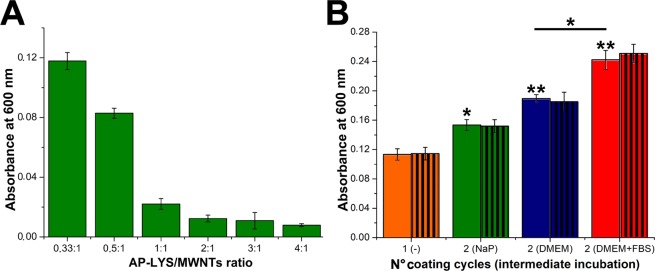
Preparation of MWNTs scaffolds in cell culture-treated polystyrene plates. (**A**) Absorbance at 600 nm of wells treated with MWNT dispersions “c” to “h”. (**B**) Absorbance at 600 nm of wells treated with MWNT dispersion “c” once (orange bars) or twice with an intermediate incubation in NaP pH 7.4 (green bars), DMEM (blue bars), DMEM containing 10% FBS (red bars). Absorbance values were measured immediately after preparation (coloured bars) and after incubation for 96 h in DMEM containing 10% FBS (striped bars). One and two asterisks indicate p < 0.05 and p < 0.02, respectively, with respect to the sample treated once with MWNT dispersion “c”.

Thus, AP-LYS-coated MWNTs was used to coat the bottom of cell culture-treated polystyrene wells, which are characterized by a hydrophilic and negatively charged surface. AP-LYS-coated MWNTs, obtained using all AP-LYS/MWNTs ratios (from 0.33:1 to 4:1), were added to polystyrene plates for 2 h in AMAC at pH 5.0. At the end of the incubation, wells were extensively washed with AMAC and the amount of bound nanotubes was measured by reading the absorbance at 600 nm (Fig. 6A).

All the MWNTs samples were able to coat the bottom of the wells but with different efficiency, depending on the AP-LYS:MWNTs ratio. The sample containing the lowest AP-LYS/MWNTs ratio (sample c) provided the highest amount of bound nanotubes. Increasing the AP-LYS/MWNTs ratio determined a considerable reduction in the amount of bound nanotubes (Fig. 6A). This result could be explained hypothesizing that AP-LYS-coated MWNTs dispersions obtained using lower amounts of AP-LYS (samples “c” and “d”) contain not-shielded hydrophobic patches which could contribute to stabilize the binding.

We reasoned that neutralizing the positive charge of the AP-LYS-coated MWNTs layer deposited at the bottom of the well could allow to add a second layer of coated nanotubes trough a layer by layer deposition process. Hence, wells coated with sample “c” were incubated in different conditions: (i) with NaP, (ii) with DMEM or (iii) with DMEM in the presence of 10% FBS. After the removal of the buffer/medium, wells were treated again with sample “c”. Figure S11 shows the aspect of wells after the deposition of the two layers. As shown in Fig. 6B the second coating step determined a further increase of the optical density, thus indicating an increase in the amount of bound nanotubes. However, the increase was modest in the wells washed with NaP before the second coating step and significant in the wells treated with DMEM.

In particular, in the wells treated with DMEM containing 10% FBS, the O.D._600nm_ were approximately doubled after the second coating step (Fig. 6B), thus indicating that FBS is particularly effective in shielding the positive charge of coated nanotubes. FBS is a very complex mixture of proteins including negatively charged proteins like albumins. These proteins could be adsorbed at the surface of the cationic AP-LYS-coated MWNTs layer, thus forming an anionic layer, which could promote the adhesion of a second layer of AP-LYS-coated MWNTs. However, we could not exclude that also specific AP-LYS/serum protein interactions contribute to binding. Very interestingly, the amount of bound nanotubes did not change after incubation of the wells for 96 h at 37 °C in DMEM containing 10% FBS, thus indicating that the AP-LYS-coated MWNTs scaffolds were stable under cell culture conditions (Fig. 6B).

**Figure 7:**
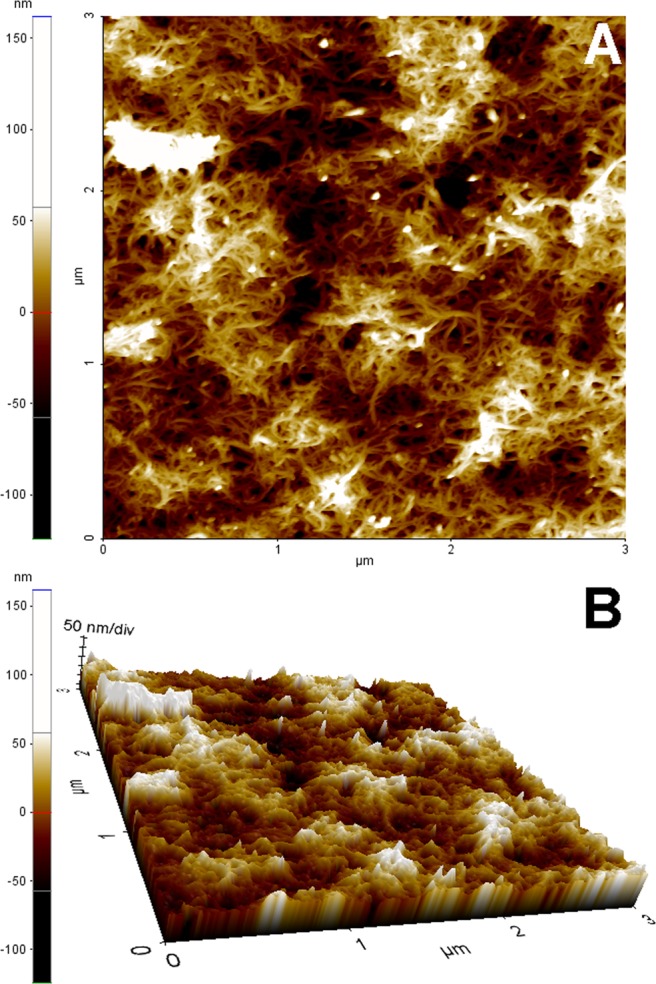
2-D (**A**) and 3-D (**B**) AFM images of the AP-LYS-coated MWNTs scaffolds prepared in the wells of a cell culture-treated polystyrene plate by the two layers deposition procedure.

The scaffolds obtained through the two-steps deposition procedure were analyzed by scanning electron microscopy and AFM. Both techniques evidenced the presence of a complex and tangled net of nanotubes (Figs 7 and Fig. S12). In particular, AFM images showed a very irregular surface with several valleys and ridges a feature usually considered well suited for the adhesion of eukaryotic cells.

Finally, two human cell lines, the cancerous HeLa and the immortalized HaCaT cell lines, were cultivated for 24 to 72 h in the wells coated through the two-layers deposition procedure. The Alamar blue cell viability assay showed that the AP-LYS-coated MWNTs scaffold did not influenced cell viability (Fig. S13). Moreover, fluorescence microscopy analysis of the cultures revealed no morphological alterations in both cell lines (Fig. S14). It can thus be concluded that the scaffolds containing AP-LYS-coated MWNTs are well suited for eukaryotic cell cultures.

## Conclusions

We have demonstrated that AP-LYS, an irreversibly denatured and chemical modified lysozyme derivative, is a very effective tool to prepare long term stable dispersions of carbon nanotubes. The proposed procedure is simple, fast and environment friendly, being carried out without using organic solvents, thus allowing an easy industrial scaling up. It is also worth remembering that hen egg lysozyme is a cheap and widely available protein. To this regard, it is interesting to compare the efficiency of native LYS and AP-LYS as CNT dispersants. Horn and colleagues, starting from a suspension containing 1 mg/ml of CNTs and 5 mg/ml native LYS, obtained a stable dispersion containing 0.15 mg/ml of CNTs and 2.2 mg/ml of native LYS^31^. Similarly, Nepal and Geckeler using an excess of native lysozyme obtained a stable dispersion containing 0.09 mg/ml of CNTs^30^. On the other hand, AP-LYS allowed to obtain long term stable dispersions containing 1 mg/ml of CNTs, using AP-LYS concentrations lower than that of CNTs. These performances are likely due to the fact that denatured AP-LYS exposes all its hydrophobic residues, whereas native lysozyme only exposes small patches of hydrophobic residues at the surface of the native fold. Likely, the intrinsic flexibility and length of the unfolded protein further contribute to favour an extended and strong interaction between AP-LYS and the carbon surface. From this point of view AP-LYS is more similar to random copolymers of amino acids (e.g. co-polymers of lysine and tryptophan^26^) than to globular proteins. However, differently from copolymers, AP-LYS has a defined weight and sequence. Apart from the direct comparison in terms of yields and effective concentrations, we want to underline that native lysozyme and AP-LYS are not alternative but complementary dispersing agents. In fact, native lysozyme maintains most of its catalytic activity after binding to CNTs^30–32^, therefore, it could be the better suited agent when lytic antibacterial activity is an added value^32^. On the other hand, when catalytic activity is not necessary or even undesirable (e.g. when the aim is to flocculate bacterial cells) AP-LYS is better suited. Furthermore, AP-LYS itself is not just a dispersing agent, but it also adds new functionalities to CNTs. The high positive charge and the presence of bioactive antimicrobial peptide-like regions make AP-LYS-coated CNTs a very effective tool for microbial cell flocculation with an efficiency similar to that of chitosan, a benchmark in the field. Moreover, we have shown that precipitation in eukaryotic cell culture media allows to prepare stable scaffolds with high surface roughness. This could be useful in the cultivation of cell lines with particular requirements, such as neurons and myocytes. We want also to underline that AP-LYS-coated CNTs could be further covalently modified by exploiting several very well-known protein-modification strategies^59^, thus expanding the potential applications of this new bioconjugate. Finally, it is worth noting that, similarly to lysozyme(s), other disulfide rich proteins – e.g. pancreatic type ribonucleases – can be reduced to obtain denatured yet soluble proteins^60^, thus suggesting that our approach could pave the way toward a new family of biohybrid materials.

## Methods

### Materials

Lysozyme from chicken egg white (purity ≥ 90%) and multi-walled carbon nanotubes (O.D × I.D. × L = 10 nm ± 1 nm × 4.5 nm ± 0.5 nm × 3–6 µm, purity ≥ 98%) were purchased by Sigma Aldrich.

*Escherichia coli* strain 25922, *Salmonella enterica Thiphimurium* strain 14028, *Enteroccoccus faecalis* strain 29212, and *Saccharomyces cerevisae* strain 26602 were obtained from ATCC.

### Preparation of MWNTs dispersions using AP-LYS

AP-LYS was prepared as previously described^36^. MWNTs dispersions were prepared in batches of 3 mL in 10 mM ammonium acetate pH 5.0 by mixing 3 mg of MWNTs powder (Sigma-Aldrich) and different AP-LYS concentrations (from 0.25 to 4 mg/mL), using a medium power tip sonicator (Sonics, Vibra cell VC505). Samples were sonicated in an ice bath for 10–60 min. The suspensions were centrifuged at 1000 g for 10 min at 4 °C. Supernatants were collected and stored at 4 °C. Supernatants were labelled “a”–“h” as detailed in Table 1.

### Characterization of the dispersions

The UV-Vis spectra of samples “a”–“h”, prepared as described in the previous section, were recorded using an UV-Vis spectrophotometer Cary 100 Scan using a 1 cm quartz cell.

Transmission electron microscopy (TEM) measurements were performed on a JEOL JEM 1011 microscope operating at an accelerating voltage of 100 KV. A droplet of the MWNTs was directly deposited on a microscope grid and analyzed.

Electrokinetic analysis and ζ-potential were carried out in folded capillary cells using a Malvern Zetasizer Nano-ZS system equipped with a 633 nm He–Ne laser. All measurements were conducted at 25 °C.

### Stability as function of pH and ionic strength

The MWNTs dispersions were diluted in different buffers: 10 mM AMAC pH 5.0, 10 mM MOPS pH 7.4 and 10 mM NaP pH 7.4, in the absence or presence of 150 mM NaCl. After 20 min incubation at room temperature the samples were centrifugated at 1000 g for 20 min at 4 °C, hence the supernatants were analyzed by UV-Vis measurements using a Nanodrop 2000 spectrophotometer (Thermo scientific). The fraction of coated MWNTs remaining dispersed after the dilution in a given buffer was calculated by the equation:

1 $${\rm{Dispersed}}\,{\rm{fraction}}\,( \% )=({\rm{O}}{\rm{.D}}{{{\rm{.}}}_{600{\rm{nm}}}}^{{\rm{buffer}}}/{\rm{O}}{\rm{.D}}{{{\rm{.}}}_{600{\rm{nm}}}}^{{\rm{AMAC}}})\times 100$$

where O.D._600nm_^buffer^ is the optical density of a nanotube dispersion diluted in the buffer and O.D._600nm_^AMAC^ is the optical density of the nanotube dispersion diluted at the same final concentration in 10 mM AMAC pH 5.0.

### Docking of lysozyme fragment 1–18

The interaction of fragment 1–18 of lysozyme with graphene and CNT surfaces was modelled by using a Monte Carlo energy minimization strategy as previously described for a MoS_2_ monolayer^37^. All calculations were performed using the ZMM-MVM molecular modelling package (ZMM Software Inc. [http://www.zmmsoft.com]) which has already proved useful for the modelling of several complexes of different nature and size^61–65^. The model complexes included lysozyme fragment 1–18 and a fragment either of a planar graphene sheet or of a carbon nanotube with an outer diameter of about 10 nm and a chirality index (73, 73) generated by the TubeASP, carbon nanotube generation applet (http://www.nanotube.msu.edu/tubeASP/). Both carbon surfaces included 2074 carbon atoms with approximate dimension ~74 Å × ~71 Å in the case of the planar surface and ~74 Å × ~65 Å in the case of the CNT fragment. Both the graphene and CNT fragments were frozen throughout the simulation process.

Initial structures of the lysozyme fragment were prepared using PyMOL (DeLano Scientific LLC, https://www.pymol.org/) and DeepView - Swiss-PdbViewer^66^. Two initial structures were modelled, a completely helical and a completely extended structure. Each structure was used to generate eight starting manually prepared complexes for each of the two carbon surfaces: fragment 1–18 was placed parallel to the surface with the termini of the peptide pointing toward either the zigzag or the armchair edges of the carbon surface, hence the peptide structure was rotated by steps of 90°. Each of the sixteen models (eight for the planar and eight for the curved surface) was used as starting point for a Monte Carlo trajectory. Trajectories were stopped when no energy decrease was observed for 1000 minimization cycles. Energy calculations included an implicit water solvation component calculated as previously described^67,68^.

For each optimized complex total peptide/carbon surface binding energies were calculated by summing the residue/carbon surface binding energies provided by the ZMM Software.

Fragment 1–18 alone was also modelled (Fig. S4B) in order to calculate a theoretical change in Gibbs free energy ΔG for the adsorption reaction:

peptide_(aq.)_ + carbon surface_(aq.)_ = peptide/carbon surface complex_(aq.)_

ΔG was calculated by the equation:

2 $$\Delta G={{\rm{E}}}_{{\rm{c}}{\rm{o}}{\rm{m}}{\rm{p}}{\rm{l}}{\rm{e}}{\rm{x}}}-{{\rm{E}}}_{{\rm{p}}{\rm{e}}{\rm{p}}{\rm{t}}{\rm{i}}{\rm{d}}{\rm{e}}}-{{\rm{E}}}_{{\rm{C}}-{\rm{s}}{\rm{u}}{\rm{r}}{\rm{f}}{\rm{a}}{\rm{c}}{\rm{e}}}$$

where E_complex_ is the energy of the minimized carbon surface/peptide complex; E_C-surface_ is the energy of the carbon surface alone; E_peptide_ is the energy of the peptide minimized in the absence of the carbon surface.

### Flocculation

Flucculation was determined as previously described^54^. *Escherichia coli* (ATCC 25922)*, Salmonella enterica Thiphimurium* (ATCC 14028)*, Enteroccoccus faecalis* (ATCC 29212)*, Saccharomyces cerevisae* (ATCC 26602) were grown in Luria-Bertani medium at 37 °C in 1 L shake flasks overnight. Growth was followed by measuring the optical density at 600 nm recorded by an UV-Vis spectrophotometer Cary 100 Scan. Cells in the stationary phase were collected by centrifugation (5000 rpm, 10 min at room temperature), washed twice with PBS, and resuspended either in 10 mM AMAC pH 5.0 or 10 mM AMAC pH 5.0 containing 150 mM NaCl at a final optical density at 600 nm of 3.2. Different concentrations of MWNT dispersions (from 0.175 to 5.6 µg/mL) were added to bacterial suspension and then the suspensions were stirred on a vortex for 2 min. The optical density at 600 nm of the supernatants was recorded after 2 and 18 h. Flocculation was expressed as the decrease of turbidity relative to the control without nanotubes and calculated by the equation:

3 $$ \% \,{\rm{flocculation}}=[1-({\rm{O}}{\rm{.D}}{{{\rm{.}}}_{600{\rm{nm}}}}^{{\rm{sample}}}/{\rm{O}}{\rm{.D}}{{{\rm{.}}}_{600{\rm{nm}}}}^{{\rm{control}}})]\times 100$$

Flocculation experiments were conducted at room temperature and each experiment was repeated three times.

The LIVE/DEAD Bacterial Viability Kit (BacLightE) was used to determine the viability of bacteria after incubation with MWNT. Staining was performed according to the manufacturer instructions. The flocs were placed on microscope slides and covered with a coverslip. Images were captured using an Olympus DP70 digital camera equipped with Olympus U-CA Magnification Changer (magnification 100×) and processed with Image Analysis Software (Olympus) for minor adjustments of brightness, contrast and color balance and for creation of merged images. Exposure times were in the range between 50 and 200 ms.

### AP-LYS-coated MWNTs binding to polystyrene plates

Fifty microliters of AP-LYS-coated MWNTs samples “c”–“h” were added to cell culture-treated polystyrene plates (96-well, Corning). After 2 h incubation at 37 °C, wells were washed with 10 mM AMAC pH 5.0 for three times and the degree of coating of polystyrene wells was measured by reading the absorbance at 600 nm in a multiwell reader (Synergy HTX Multi-Mode Reader-BIOTEK). All the absorbance values were corrected subtracting the absorbance of untreated wells.

### Layer by layer deposition of AP-LYS-coated MWNTs

Polystyrene wells treated with AP-LYS-coated MWNT sample “c” as described in the previous section, after extensive washes with AMAC buffer (10 mM, pH 5.0), were incubated for 60 min at 37 °C in different conditions: (i) with 10 mM NaP, pH 7.4; (ii) with DMEM; (iii) with DMEM in the presence of 10% FBS. After removal of NaP and DMEM, wells were incubated again with 50 µL of sample “c” for 1 h at 37 °C. The degree of coating of polystyrene wells was measured by reading the absorbance at 600 nm at the end of the preparation and after 96 h incubation at 37 °C in DMEM containing 10% FBS.

### Cell cultures

The Alamar Blue assay was used to assess cell viability based on the reduction potential of metabolically active cells. Human immortalized keratinocytes (HaCaT) and human cancer epithelial cells (HeLa) were cultivated in the wells coated with two layers of MWNTs (sample c) in complete medium (DMEM containing 10% FBS, 2 mM L-glutamine and antibiotics) at 37 °C in a humidified atmosphere containing 5% CO_2_. At the end of incubation, AlamarBlue® reagent (Invitrogen) was added in each well and incubated for 3 h at 37 °C. The fluorescence intensity was measured at an emission wavelength of 585 nm and an excitation wavelength of 570 nm using a plate reader (Synergy HTX Multi- Mode Reader-BIOTEK). Cell survival was expressed as the percentage of viable cells grown on the nanotube scaffolds compared to controls. Each sample was tested in three independent analyses, each carried out in triplicate. Cell viability was expressed as mean values ± SD. Significance was determined by Student’s t-test at a significance level of 0.05. In order to analyze the morphology of HaCaT and HeLa cells grown on the CNT scaffolds the cells were stained with the viable, cell permeable fluorescent stain fluorescein diacetate (FDA). FDA stock solution was prepared in acetone at 5 mg/mL. Cells were seeded in 96-well plates coated by MWNTs dispersion (sample c) at a density of 1 × 104/well and then grown at 37 °C for 24 h. Cells were treated with FDA (1 µg/mL) in DMEM without FBS for 5 minutes in the dark. After removing the staining solution, DMEM was added to the cells. Cells were analyzed by a Leica DMi8 fluorescence microscope (magnification 20×) using a FITC filter. Typical acquisition time was 100 ms. The images were captured using a Leica DFC 310 FX digital camera and processed by using the analysis software supplied by the manufacturer.

### SEM and AFM

Microbial flocs were imaged after deposition by casting of about 3 µl of solution on a gold surface and drying at room temperature as described^69^. Usually, biological materials are metalized prior to SEM imaging, however, metallization may cover nanometric features, as the presence of nanotubes on the flocs. Therefore, metallization was not performed and conductivity was provided by the presence of the nanotube net. AP-LYS-coated MWNTs scaffolds, prepared at the bottom of polystyrene wells as described in section 4.8, were imaged without removing the scaffolds from the plastic support. Polystyrene fragments of 3–4 mm were cut from the bottom of the wells and imaged without any further treatment.

SEM images were performed at 5 kV accelerating voltage and 20 µm wide aperture by a Field Emission Scanning Electron Microscope (Carl Zeiss NTS GmbH 1500 Raith FESEM). Secondary Emission (SE) and InLens detectors were used.

AFM images were obtained by a XE-100 AFM (Park Systems). Surface imaging was obtained in non-contact mode using silicon/aluminium coated cantilevers (SSS-NCHR 10 M; Park Systems; tip radius less then 5 nm) 125 μm long with a typical resonance frequency of 330 kHz and nominal force constant of 42 N/m. The scan frequency was typically 0.5 Hz per line and the images have 512 × 512 pixel. When necessary, the AFM images were processed by flattening, in order to remove the background slope, and the contrast and brightness were adjusted.

## Supplementary information

Supplementary figures

## Data Availability

The experimental data and the Monte Carlo models generated and analysed during the current study are available from the corresponding authors on reasonable request.

## References

[1] Xu Q (2019). Function-driven engineering of 1D carbon nanotubes and 0D carbon dots: mechanism, properties and applications. Nanoscale.

[2] Jacobs CB, Peairs MJ, Venton BJ (2010). Review: Carbon nanotube based electrochemical sensors for biomolecules. Anal. Chim. Acta.

[3] Wang J, Lin Y (2008). Functionalized carbon nanotubes and nanofibers for biosensing applications. TrAC Trends Anal. Chem..

[4] Vashist SK, Zheng D, Al-Rubeaan K, Luong JHT, Sheu F-S (2011). Advances in carbon nanotube based electrochemical sensors for bioanalytical applications. Biotechnol. Adv..

[5] Dhanabalan SC, Dhanabalan B, Chen X, Ponraj JS, Zhang H (2019). Hybrid carbon nanostructured fibers: stepping stone for intelligent textile-based electronics. Nanoscale.

[6] Mahajan S (2018). Functionalized carbon nanotubes as emerging delivery system for the treatment of cancer. Int. J. Pharm..

[7] Mishra V, Kesharwani P, Jain NK (2018). Biomedical Applications and Toxicological Aspects of Functionalized Carbon Nanotubes. Crit. Rev. Ther. Drug Carr. Syst..

[8] Pérez-López B, Merkoçi A (2012). Carbon nanotubes and graphene in analytical sciences. Microchim. Acta.

[9] Pok S (2014). Biocompatible Carbon Nanotube–Chitosan Scaffold Matching the Electrical Conductivity of the Heart. ACS Nano.

[10] Resende R (2012). Carbon nanotube interaction with extracellular matrix proteins producing scaffolds for tissue engineering. Int. J. Nanomedicine.

[11] Fabbro A, Bosi S, Ballerini L, Prato M (2012). Carbon nanotubes: Artificial nanomaterials to engineer single neurons and neuronal networks. ACS Chem. Neurosci..

[12] Vaisman L, Wagner HD, Marom G (2006). The role of surfactants in dispersion of carbon nanotubes. Adv. Colloid Interface Sci..

[13] Borzooeian Z, Safavi A, Sheikhi MH, Aminlari M, Doroodmand MM (2010). Preparation and investigation on properties of lysozyme chemically bonded to single-walled carbon nanotubes. J. Exp. Nanosci..

[14] Zhu W, Minami N, Kazaoui S, Kim Y (2004). π-Chromophore-functionalized SWNTs by covalent bonding: substantial change in the optical spectra proving strong electronic interaction. J. Mater. Chem..

[15] Karousis N, Tagmatarchis N, Tasis D (2010). Current Progress on the Chemical Modification of Carbon Nanotubes. Chem. Rev..

[16] Zhao Y-L, Stoddart JF (2009). Noncovalent Functionalization of Single-Walled Carbon Nanotubes. Acc. Chem. Res..

[17] Sprafke JK, Stranks SD, Warner JH, Nicholas RJ, Anderson HL (2011). Noncovalent Binding of Carbon Nanotubes by Porphyrin Oligomers. Angew. Chemie Int. Ed..

[18] Yang W (2013). Surface functionalization of carbon nanomaterials by self-assembling hydrophobin proteins. Biopolymers.

[19] Zhou Y, Fang Y, Ramasamy R (2019). Non-Covalent Functionalization of Carbon Nanotubes for Electrochemical Biosensor Development. Sensors.

[20] Calvaresi M, Zerbetto F (2013). The Devil and Holy Water: Protein and Carbon Nanotube Hybrids. Acc. Chem. Res..

[21] Zheng M (2003). DNA-assisted dispersion and separation of carbon nanotubes. Nat. Mater..

[22] Sanchez-Pomales Germarie, Pagan-Miranda Coral, Santiago-Rodriguez Lenibel, R. Carlos (2010). DNA-Wrapped Carbon Nanotubes: From Synthesis to Applications. Carbon Nanotubes.

[23] Ezhil Vilian AT, Chen SM, Lou BS (2014). A simple strategy for the immobilization of catalase on multi-walled carbon nanotube/poly (L-lysine) biocomposite for the detection of H2O2 and iodate. Biosens. Bioelectron..

[24] De Leo F, Magistrato A, Bonifazi D (2015). Interfacing proteins with graphitic nanomaterials: from spontaneous attraction to tailored assemblies. Chem. Soc. Rev..

[25] Tomásio SM, Walsh TR (2009). Modeling the Binding Affinity of Peptides for Graphitic Surfaces. Influences of Aromatic Content and Interfacial Shape. J. Phys. Chem. C.

[26] Salzmann CG, Ward MAH, Jacobs RMJ, Tobias G, Green MLH (2007). Interaction of tyrosine-, tryptophan-, and lysine-containing polypeptides with single-wall carbon nanotubes and its relevance for the rational design of dispersing agents. J. Phys. Chem. C.

[27] Wang S (2003). Peptides with selective affinity for carbon nanotubes. Nat. Mater..

[28] Xie H, Becraft EJ, Baughman RH, Dalton AB, Dieckmann GR (2008). Ranking the affinity of aromatic residues for carbon nanotubes by using designed surfactant peptides. J. Pept. Sci..

[29] Ge C (2011). Binding of blood proteins to carbon nanotubes reduces cytotoxicity. Proc. Natl. Acad. Sci. USA.

[30] Nepal D, Geckeler KE (2006). PH-sensitive dispersion and debundling of single-walled carbon nanotubes: Lysozyme as a tool. Small.

[31] Horn DW, Tracy K, Easley CJ, Davis VA (2012). Lysozyme dispersed single-walled carbon nanotubes: Interaction and activity. J. Phys. Chem. C.

[32] Horn DW (2013). Dispersion state and fiber toughness: Antibacterial lysozyme-single walled carbon nanotubes. Adv. Funct. Mater..

[33] Piscitelli A (2017). Applications of Functional Amyloids from Fungi: Surface Modification by Class I Hydrophobins. Biomolecules.

[34] Pennacchio A, Cicatiello P, Notomista E, Giardina P, Piscitelli A (2018). New clues into the self-assembly of Vmh2, a basidiomycota class I hydrophobin. Biol. Chem..

[35] Gravagnuolo AM (2016). Class I Hydrophobin Vmh2 Adopts Atypical Mechanisms to Self-Assemble into Functional Amyloid Fibrils. Biomacromolecules.

[36] Siepi M (2017). Modified denatured lysozyme effectively solubilizes fullerene c60 nanoparticles in water. Nanotechnology.

[37] Siepi M (2017). Production of biofunctionalized MoS _2_ flakes with rationally modified lysozyme: a biocompatible 2D hybrid material. 2D Mater..

[38] Kojima M (2011). Dispersion of single-walled carbon nanotubes modified with poly-l-tyrosine in water. Nanoscale Res. Lett..

[39] O’Brien RW, Midmore BR, Lamb A, Hunter RJ (1990). Electroacoustic studies of moderately concentrated colloidal suspensions. Faraday Discuss. Chem. Soc..

[40] Hanaor D, Michelazzi M, Leonelli C, Sorrell CC (2012). The effects of carboxylic acids on the aqueous dispersion and electrophoretic deposition of ZrO2. J. Eur. Ceram. Soc..

[41] Kelly SM, Jess TJ, Price NC (2005). How to study proteins by circular dichroism. Biochim. Biophys. Acta - Proteins Proteomics.

[42] Reiersen H, Rees AR (2000). Trifluoroethanol may form a solvent matrix for assisted hydrophobic interactions between peptide side chains. Protein Eng..

[43] Carlier L (2015). Investigating the role of GXXXG motifs in helical folding and self-association of plasticins, Gly/Leu-rich antimicrobial peptides. Biophys. Chem..

[44] Lequin O (2006). Dermaseptin S9, an α-Helical Antimicrobial Peptide with a Hydrophobic Core and Cationic Termini^†^. Biochemistry.

[45] Wang L, Wang D, Li F (2014). Insight into the structures of the second and fifth transmembrane domains of Slc11a1 in membrane mimics. J. Pept. Sci..

[46] Di Natale G (2010). Membrane Interactions and Conformational Preferences of Human and Avian Prion N-Terminal Tandem Repeats: The Role of Copper(II) Ions, pH, and Membrane Mimicking Environments. J. Phys. Chem. B.

[47] Poblete H, Miranda-Carvajal I, Comer J (2017). Determinants of Alanine Dipeptide Conformational Equilibria on Graphene and Hydroxylated Derivatives. J. Phys. Chem. B.

[48] Gregory J, O’Melia CR (1989). Fundamentals of flocculation. Crit. Rev. Environ. Control.

[49] Yang R, Li H, Huang M, Yang H, Li A (2016). A review on chitosan-based flocculants and their applications in water treatment. Water Res..

[50] Pal S, Mal D, Singh RP (2006). Synthesis, characterization and flocculation characteristics of cationic glycogen: A novel polymeric flocculant. Colloids Surfaces A Physicochem. Eng. Asp..

[51] Yang Z (2014). Flocculation of Escherichia coli using a quaternary ammonium salt grafted carboxymethyl chitosan flocculant. Environ. Sci. Technol..

[52] Hughes J, Ramsden DK, Symes KC (1990). The flocculation of bacteria using cationic synthetic flocculants and chitosan. Biotechnol. Tech..

[53] Strand SP, Vandvik MS, Vårum KM, Østgaard K (2001). Screening of chitosans and conditions for bacterial flocculation. Biomacromolecules.

[54] Strand SP, Nordengen T, Østgaard K (2002). Efficiency of chitosans applied for flocculation of different bacteria. Water Res..

[55] Strand SP, Varum KM, Østgaard K (2003). Interactions between Chitosans and Bacteria: Flocculation and Adhesion. Colloids Surfaces B Biointerfaces.

[56] Pizzo E, Cafaro V, Di Donato A, Notomista E (2018). Cryptic Antimicrobial Peptides: Identification Methods and Current Knowledge of their Immunomodulatory Properties. Curr. Pharm. Des..

[57] Pane K (2017). Antimicrobial potency of cationic antimicrobial peptides can be predicted from their amino acid composition: Application to the detection of “cryptic” antimicrobial peptides. J. Theor. Biol..

[58] Bosi S, Fabbro A, Ballerini L, Prato M (2013). Carbon nanotubes: A promise for nerve tissue engineering?. Nanotechnol. Rev..

[59] Boutureira O, Bernardes GJL (2015). Advances in Chemical Protein Modification. Chem. Rev..

[60] Notomista E (1999). Effective expression and purification of recombinant onconase, an antitumor protein. FEBS Lett..

[61] Donadio G (2015). The toluene o-xylene monooxygenase enzymatic activity for the biosynthesis of aromatic antioxidants. PLoS One.

[62] De Rosa M (2013). Novel promising linezolid analogues: Rational design, synthesis and biological evaluation. Eur. J. Med. Chem..

[63] Notomista E, Cafaro V, Bozza G, Di Donato A (2009). Molecular Determinants of the Regioselectivity of Toluene/o-Xylene Monooxygenase from Pseudomonas sp. Strain OX1. Appl. Environ. Microbiol..

[64] Notomista E (2011). Tuning the specificity of the recombinant multicomponent toluene o-xylene monooxygenase from Pseudomonas sp. strain OX1 for the biosynthesis of tyrosol from 2-phenylethanol. Appl. Environ. Microbiol..

[65] Zanfardino A (2010). Isolation of an Escherichia coli K4 kfoC mutant over-producing capsular chondroitin. Microb. Cell Fact..

[66] Guex N, Peitsch MC (1997). SWISS-MODEL and the Swiss-PdbViewer: An environment for comparative protein modeling. Electrophoresis.

[67] Weiner SJ (1984). A new force field for molecular mechanical simulation of nucleic acids and proteins. J. Am. Chem. Soc..

[68] Lazaridis T, Karplus M (1999). Effective energy function for proteins in solution. Proteins Struct. Funct. Genet..

[69] Rea I (2016). Bioengineered Silicon Diatoms: Adding Photonic Features to a Nanostructured Semiconductive Material for Biomolecular Sensing. Nanoscale Res. Lett..

