# Supplementary material for: Denatured lysozyme-coated carbon nanotubes: a versatile biohybrid material
Source: Sci Rep. 2019 Nov 12;9:16643. doi: 10.1038/s41598-019-52701-9 (PMC6851173; doi:10.1038/s41598-019-52701-9)
Supplement: Supplementary file 1 — Supplementary figures [file 41598_2019_52701_MOESM1_ESM.pdf]

Denatured lysozyme-coated carbon nanotubes: a versatile biohybrid material.

Marialuisa Siepi[a], Giuliana Donadio[a], Principia Dardano[b], Luca De Stefano[b,*], Daria Maria Monti[c,*], Eugenio Notomista[a,*]

[a] Department of Biology University of Naples Federico II, Via Cintia, 80126, Naples, Italy.

[b] Institute for Microelectronics and Microsystems, Unit of Naples, National Research Council, Via P. Castellino 111, 80131, Napoli, Italy

[c] Department of Chemical Sciences University of Naples Federico II, Via Cintia, 80126, Naples, Italy.

*Corresponding authors (luca.destefano@na.imm.cnr.it; mdmonti@unina.it; eugenio.notomista@unina.it)

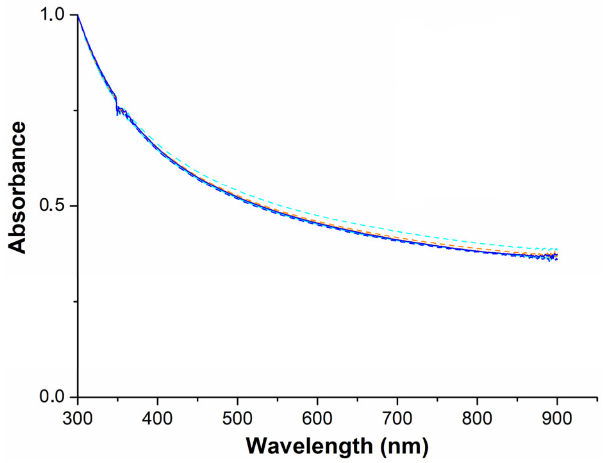

Figure S1. UV-Vis spectra of MWNTs samples "c" (cyan), "e" (orange) and "g" (blue) as prepared (solid line) and after 26 months at 4°C (dashed line).

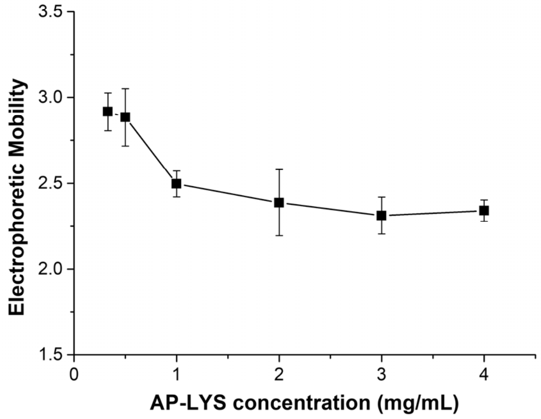

Figure S2. Electrophoretic mobility of AP-LYS-coated MWNTs dispersions.

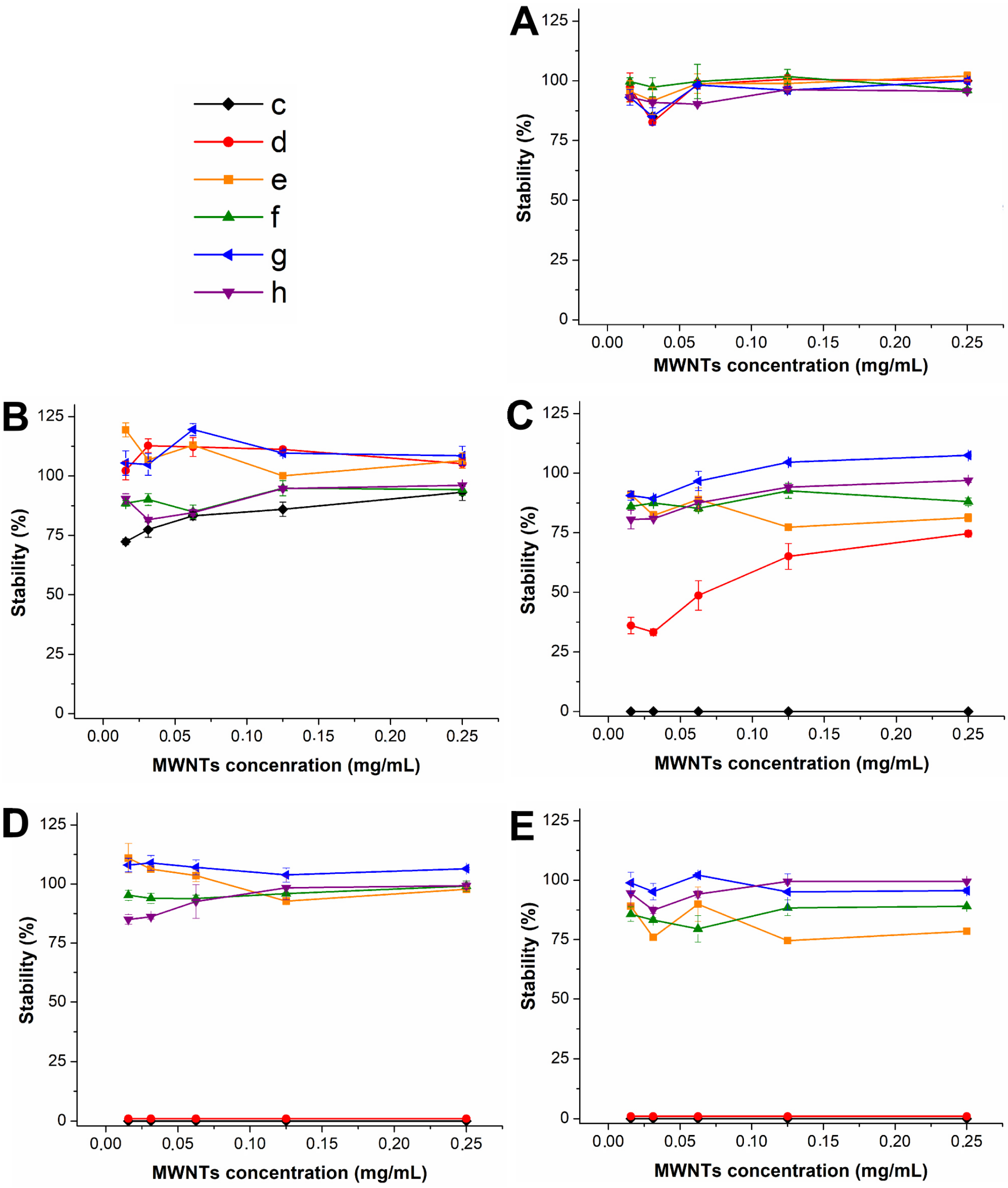

Figure S3. MWNTs stability as function of buffer, pH and ionic strength. Absorbance at 600 nm was measured after incubation in AMAC pH 5.0 in the presence of 150 mM NaCl (A), MOPS pH 7.4 (B), MOPS pH 7.4 in the presence of 150 mM NaCl (C), NaP pH 7.4 (D) and NaP pH 7.4 in the presence of 150 mM NaCl (E). MWNT dispersions "c" to "h" correspond to AP-LYS:MWNTs ratios = 0.33:1 (black line), 0.5:1 (red line), 1:1 (orange line), 2:1 (green line), 3:1 (blue line) and 4:1 (purple line), respectively.

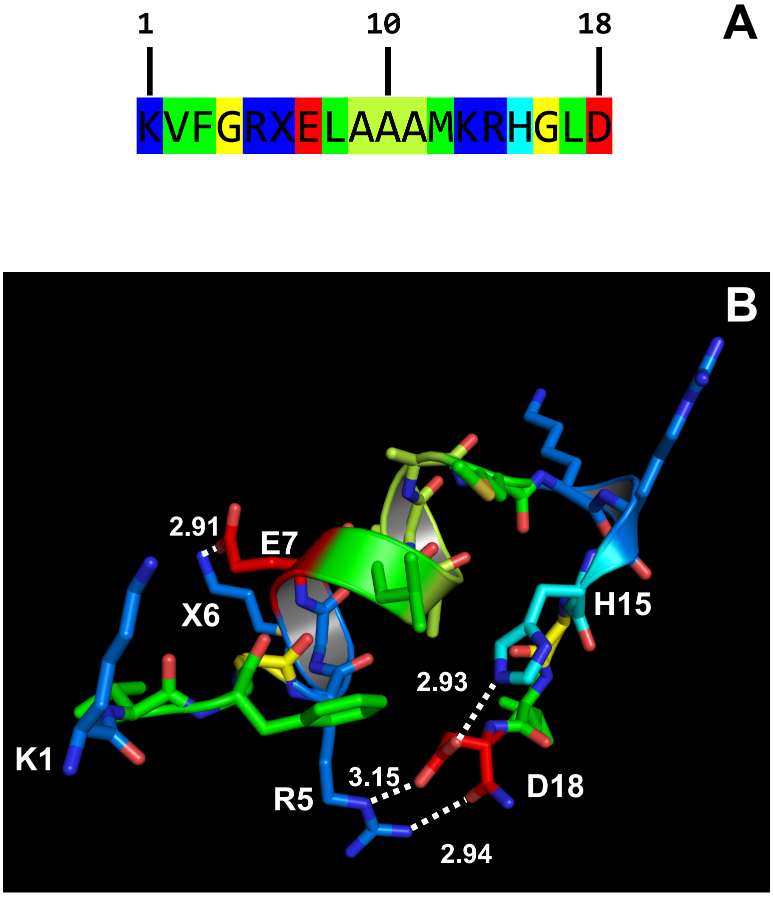

Figure S4. Sequence (A) and minimized structure in water (B) of lysozyme fragment 1-18. A) Residues are colored according to amino acids properties (green, hydrophobic; light green, alanine; blue, positively charged; red, negatively charged; cyan, histidine; yellow, glycine). B) Residues are shown as sticks colored by atom type: nitrogen, blue; oxygen, red; sulfur, dark yellow; carbon atoms are colored according to amino acids properties as in panel A. Hydrogen bonds are shown as white dotted lines and corresponding distances are in Å. In both panels, X = aminopropyl-cysteine.

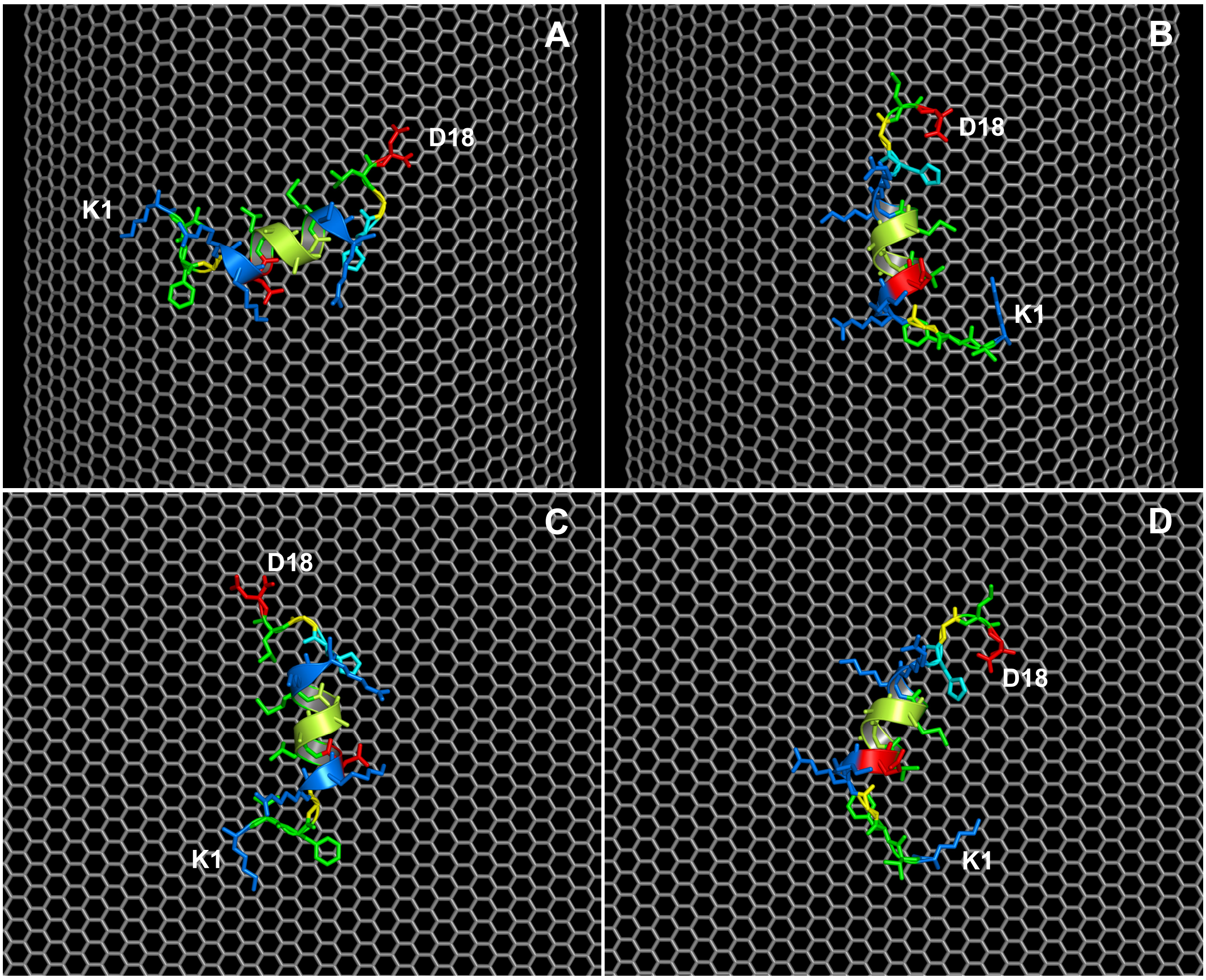

Figure S5. Docking of fragment 1-18 of hen egg lysozyme onto the surface of a CNT and a graphene monolayer. Panels A and B show models 1 and 2, respectively, on the surface of a CNT. Panels C and D show models 1 and 2, respectively, on the surface of a graphene layer. The protein fragment is shown as cartoon and sticks to highlight the secondary structure of the peptide and the side chains contacting the carbon surface. Residues are colored according to their properties: green, hydrophobic; light green, alanine; blue, positively charged; red, negatively charged; cyan, histidine; yellow, glycine. Labels are shown only for the first (K1) and the last (D18) residue. Carbon atoms of the CNT and the graphene layer are shown as gray sticks.

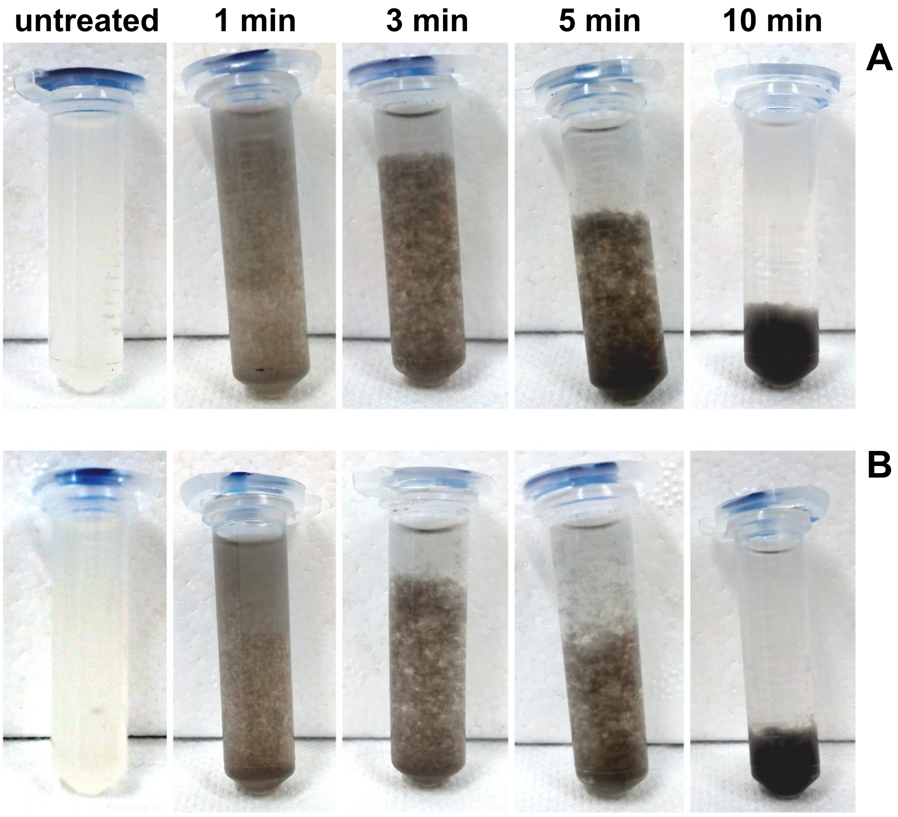

Figure S6. Flocculation of *E. coli* cultures induced by the addition of AP-LYS-coated MWNTs dispersions "c" (A) and "e" (B) with AP-LYS:MWNTs ratios = 0.33:1 and 1:1, respectively. Pictures were taken at increasing times (as indicated) after the addition of AP-LYS-coated MWNTs at a final MWNT concentration of 5.6 µg/ml. The pictures on the left show *E. coli* cultures before the addition of the MWNTs dispersions.

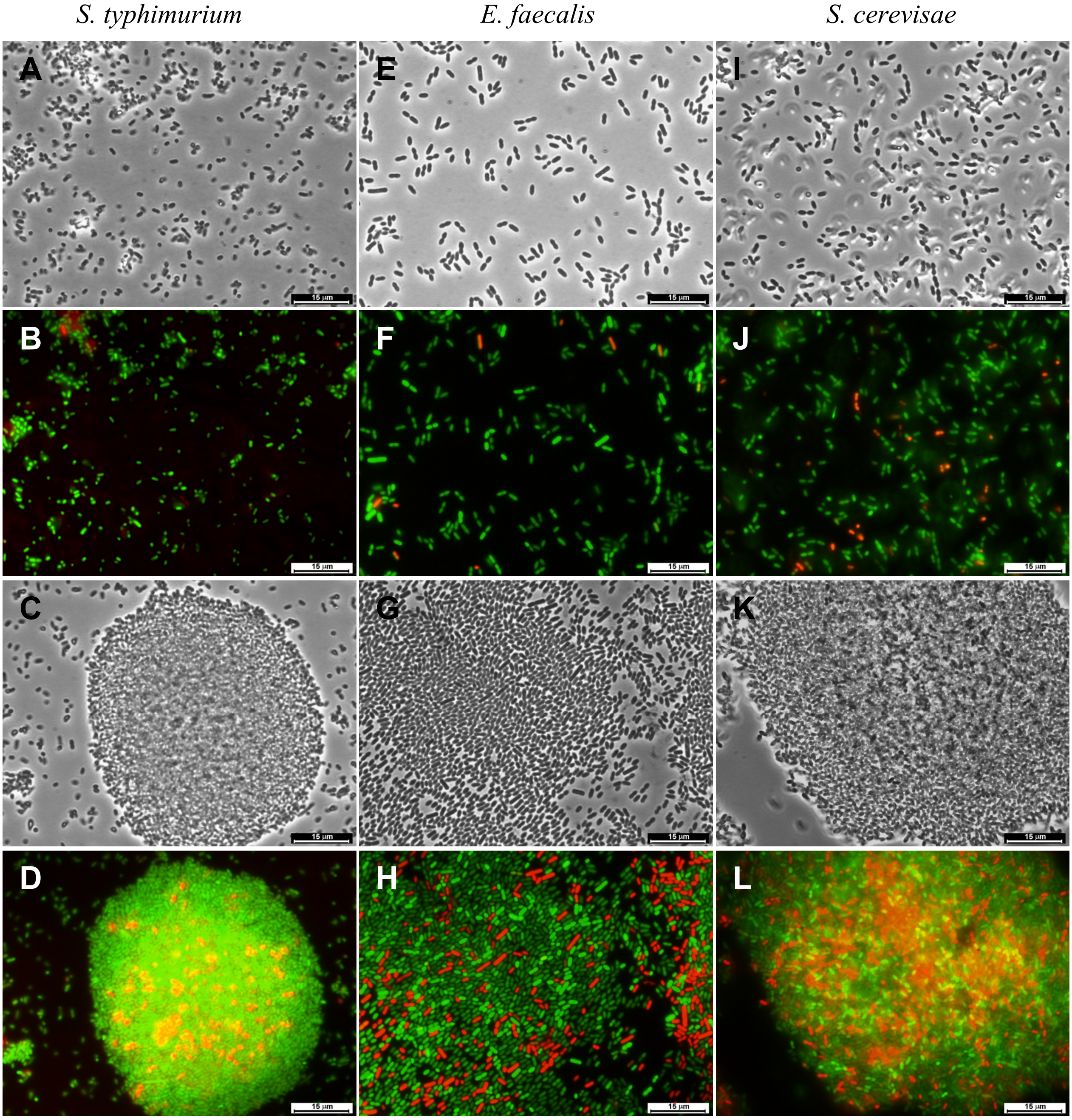

Figure S7. Live/Dead staining of *S. typhimurium* (A-D), *E. faecalis* (E-H) and *S. cerevisae* (I-L) cultures before (A, B, E, F, I, J) and 2 hours after (C, D, G, H, K, L) the addition of AP-Lys-coated MWNTs dispersion "c" (final nanotube concentration = 5.6 μg/mL). Panels A, E, I, C, G and K show phase contrast images. Panels B, F, J, D, H and L show fluorescence images obtained merging pictures taken using a FITC filter (green) and a Rhodamine filter (red).

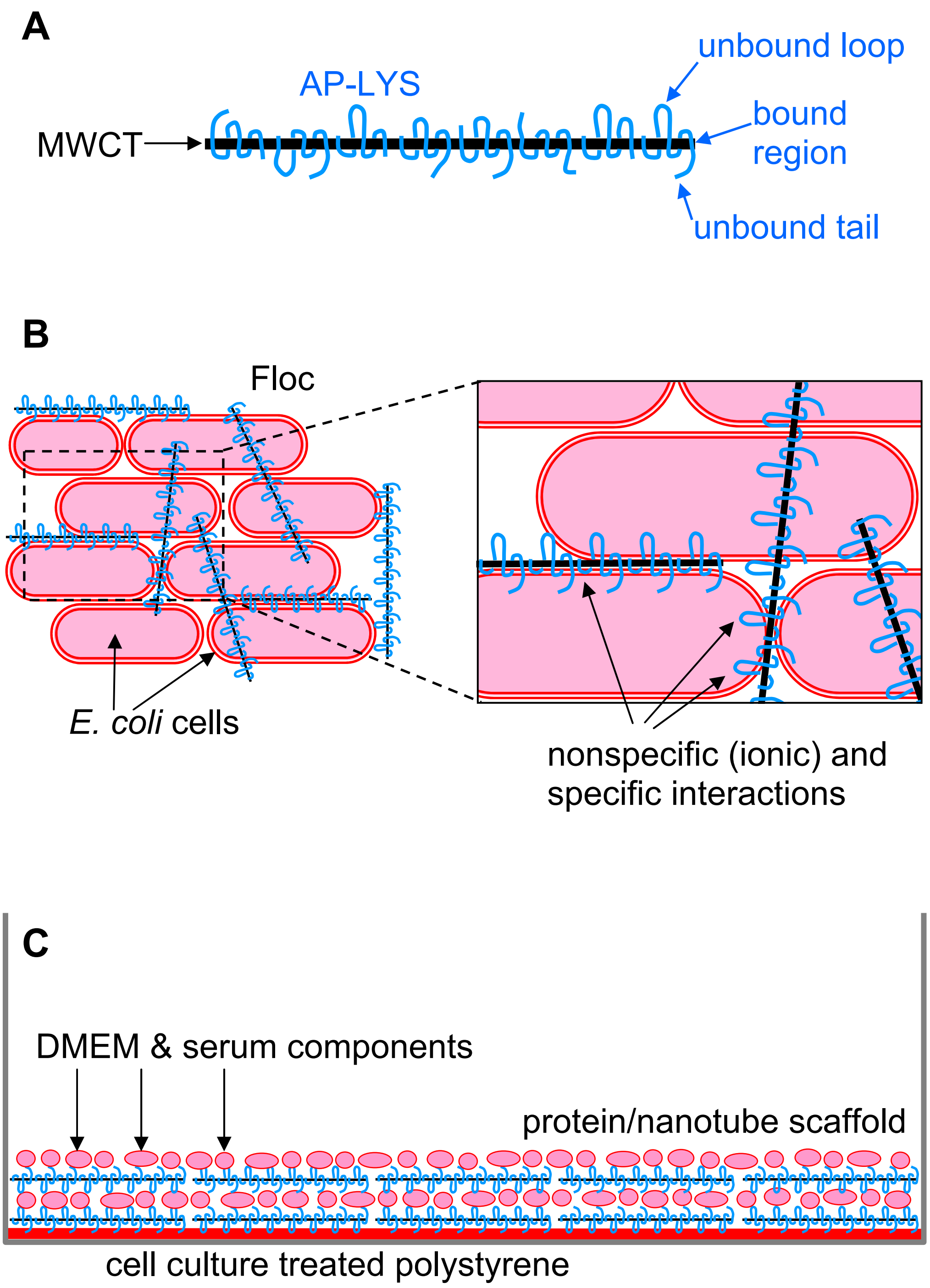

Figure S8. Schematic drawing of AP-LYS-coated MWNTs (A), *E. coli* flocs induced by the addition of AP-LYS-coated MWNTs (B), and MWNTs scaffolds deposited at the bottom of a cell culture-treated polystyrene well through the layer by layer procedure described in sections 2.4 and 4.7 (C). The interaction of AP-LYS-coated MWNTs with *E. coli* cells (B) and with chemically modified polystyrene or serum components (C) is, likely, nonspecific being driven by the electrostatic attraction between the highly cationic AP-LYS-coated MWNTs and the negatively charged surfaces of bacterial cells, treated polystyrene and serum proteins. However, specific interactions between AP-LYS and bacterial cell surface or serum proteins cannot be excluded.

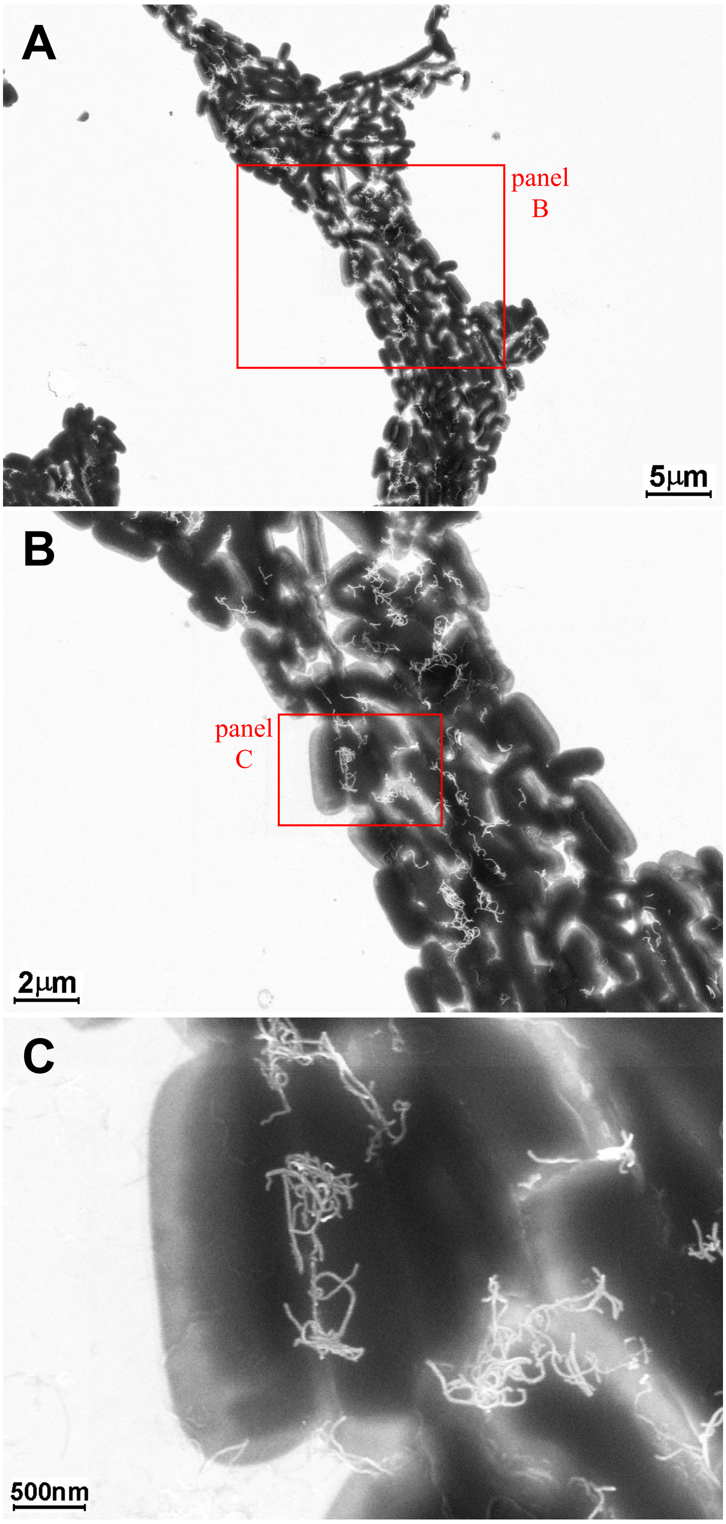

Figure S9. SEM analysis of a small floc of *E. coli* cells treated with AP-LYS-coated MWNTs dispersion “c” (AP-LYS:MWNTs ratio = 0.33:1). Images were obtained using an InLens detector. MWNTs are clearly visible in panels B and C as light strings.

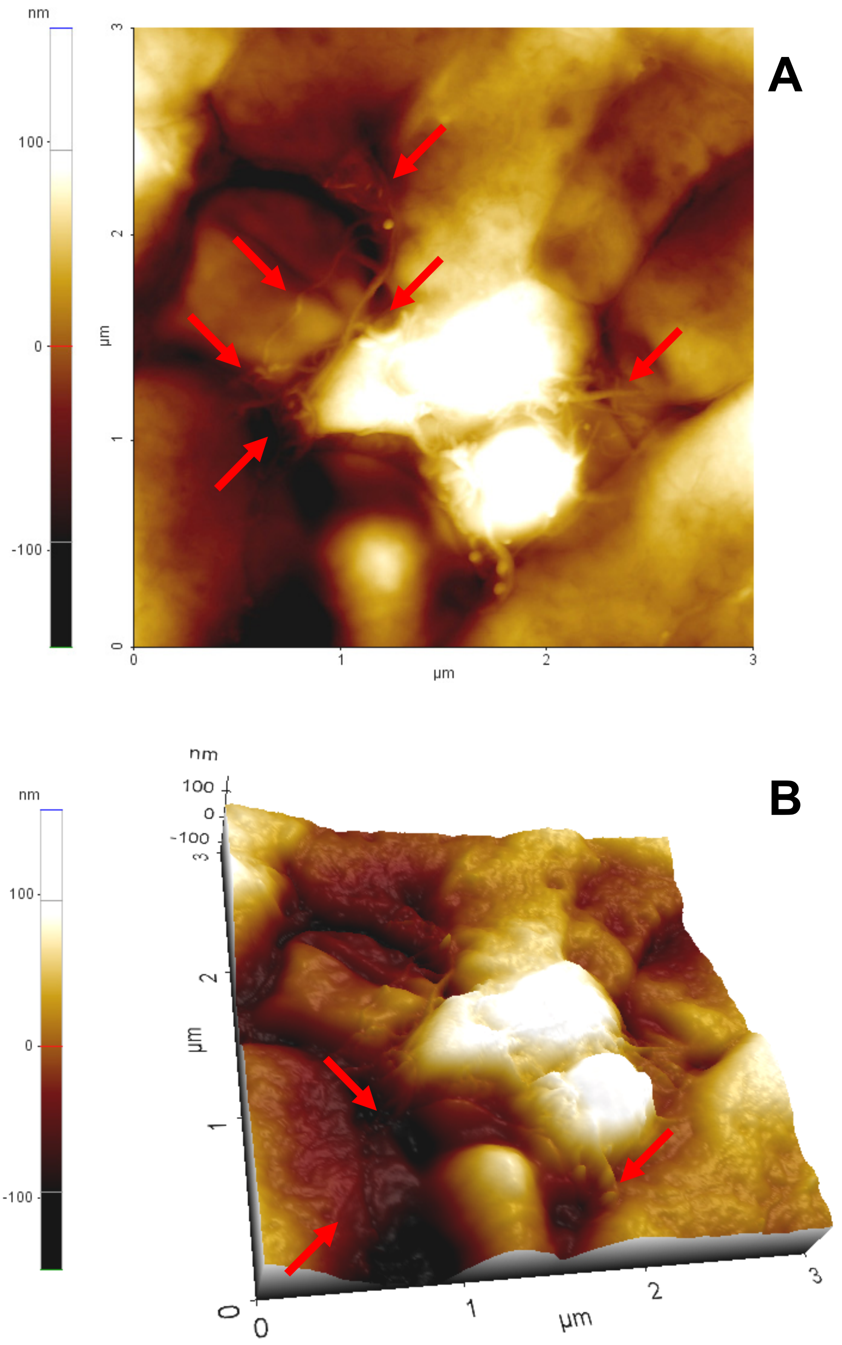

Figure S10. 2-D (A) and 3-D (B) AFM images of a floc of *E. coli* cells treated with AP-LYS-coated MWNTs dispersion "c" (AP-LYS:MWNTs ratio = 0.33:1). Red arrows show the position of MWNTs.

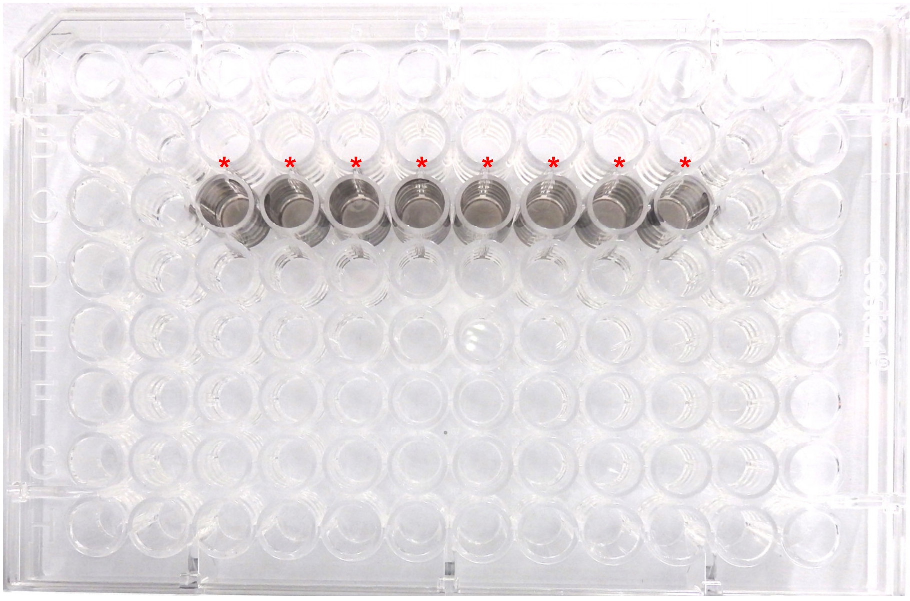

Figure S11. Picture of AP-LYS-coated MWNTs scaffolds prepared in a cell culture-treated polystyrene plate by the layer by layer deposition procedure. Treated wells, indicated by the red stars, are clearly distinguishable from untreated ones.

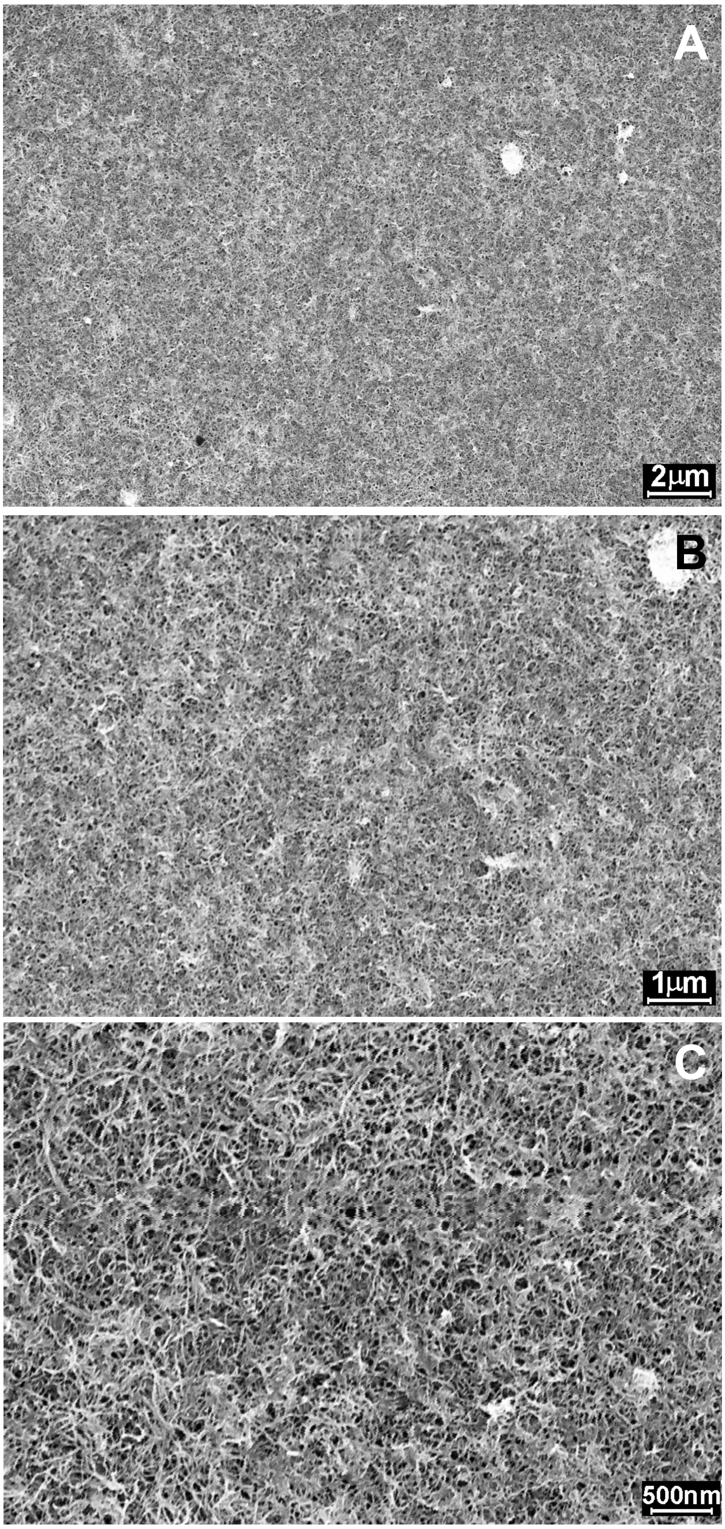

Figure S12. SEM images of the AP-LYS-coated MWNTs scaffolds prepared in the wells of a cell culture-treated polystyrene plate by the two layers deposition procedure.

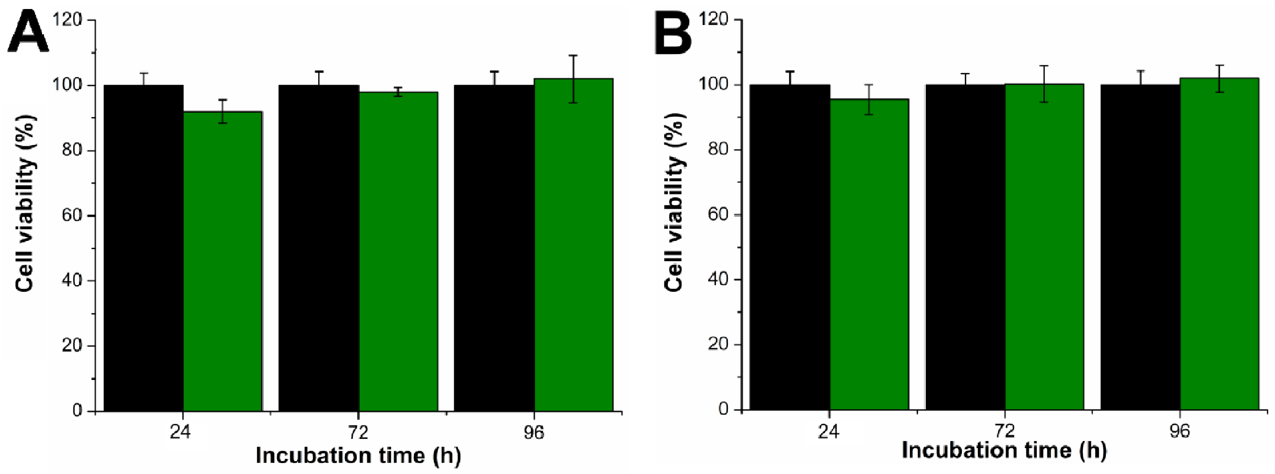

Figure S13. Alamar blue cell viability assay performed on HaCaT (A) and HeLa (B) cells grown in cell culture-treated polystyrene wells without (black bars) or with (green bars) AP-LYS-coated MWNTs scaffolds prepared by the two layers deposition procedure described in section 4.7 of the accompaining paper. Error bars correspond to the SD values of three independent experiments carried out with triplicate determinations.

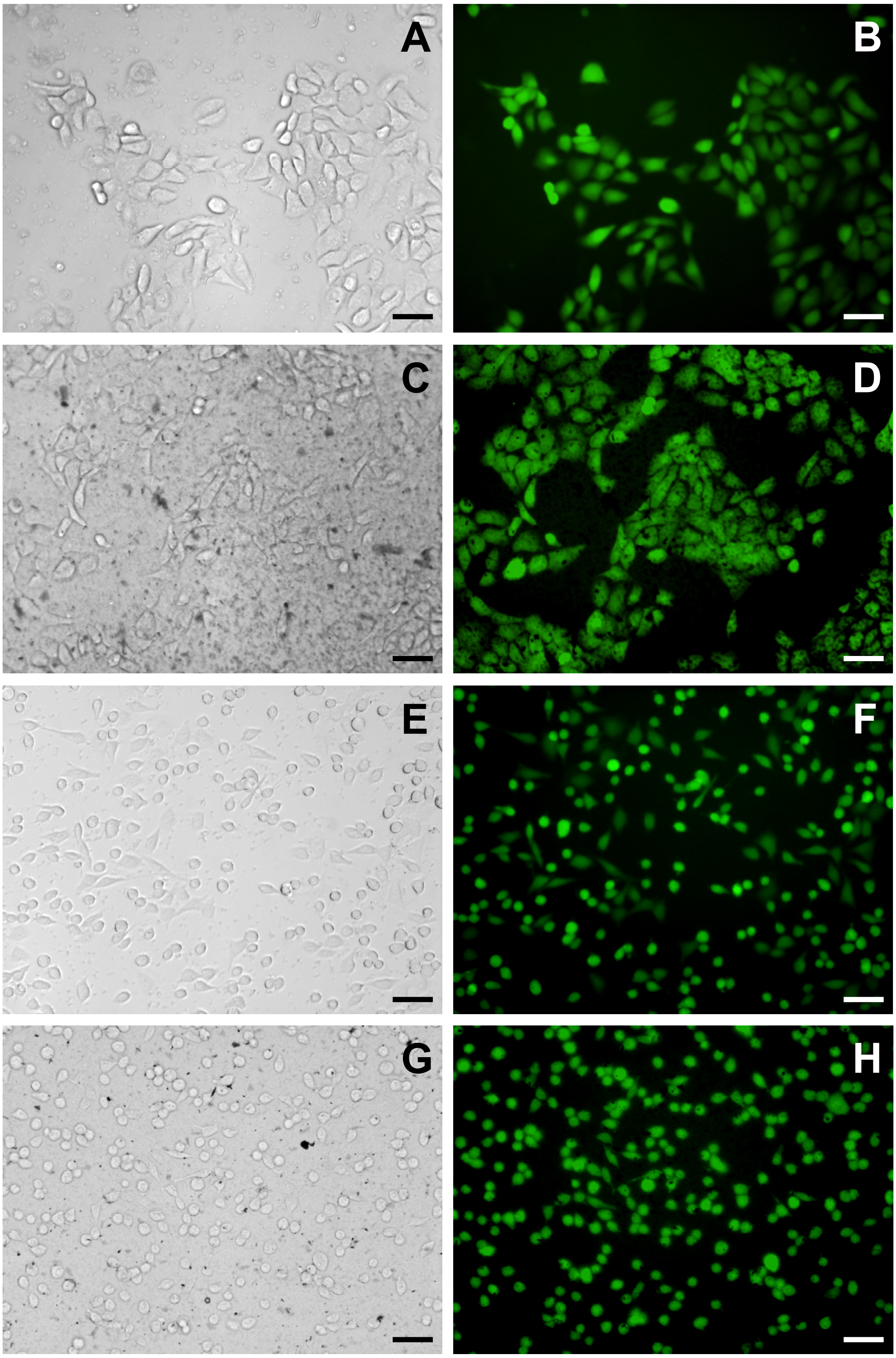

Figure S14. HaCaT (A-D) and HeLa (E-H) cells grown in cell culture-treated polystyrene wells with (C, D, G, H) or without (A, B, E, F) AP-LYS-coated MWNTs scaffolds prepared by the two layers deposition procedure. Panels A, C, E and G show phase contrast images, whereas, panels B, D, F and H show the corresponding fluorescence images. In all the panels the scale bar corresponds to 50 μm.
